# Molecular species identification of Central European ground beetles (Coleoptera: Carabidae) using nuclear rDNA expansion segments and DNA barcodes

**DOI:** 10.1186/1742-9994-7-26

**Published:** 2010-09-13

**Authors:** Michael J Raupach, Jonas J Astrin, Karsten Hannig, Marcell K Peters, Mark Y Stoeckle, Johann-Wolfgang Wägele

**Affiliations:** 1 Zoologisches Forschungsmuseum Alexander Koenig, Adenauerallee 160-162, 53113 Bonn, Germany; 2 Senckenberg am Meer, Deutsches Zentrum für Marine Biodiversitätsforschung, Südstrand 44, 26382 Wilhelmshaven, Germany; 3 Dresdener Straße 6, 45731 Waltrop, Germany; 4 The Rockefeller University, 1230 York Avenue, New York NY 10065, UK

## Abstract

**Background:**

The identification of vast numbers of unknown organisms using DNA sequences becomes more and more important in ecological and biodiversity studies. In this context, a fragment of the mitochondrial cytochrome *c *oxidase I (COI) gene has been proposed as standard DNA barcoding marker for the identification of organisms. Limitations of the COI barcoding approach can arise from its single-locus identification system, the effect of introgression events, incomplete lineage sorting, numts, heteroplasmy and maternal inheritance of intracellular endosymbionts. Consequently, the analysis of a supplementary nuclear marker system could be advantageous.

**Results:**

We tested the effectiveness of the COI barcoding region and of three nuclear ribosomal expansion segments in discriminating ground beetles of Central Europe, a diverse and well-studied invertebrate taxon. As nuclear markers we determined the 18S rDNA: V4, 18S rDNA: V7 and 28S rDNA: D3 expansion segments for 344 specimens of 75 species. Seventy-three species (97%) of the analysed species could be accurately identified using COI, while the combined approach of all three nuclear markers provided resolution among 71 (95%) of the studied Carabidae.

**Conclusion:**

Our results confirm that the analysed nuclear ribosomal expansion segments in combination constitute a valuable and efficient supplement for classical DNA barcoding to avoid potential pitfalls when only mitochondrial data are being used. We also demonstrate the high potential of COI barcodes for the identification of even closely related carabid species.

## Background

In times of climate change and massive habitat destruction, the reliable identification of species represents a pivotal component for biodiversity studies and conservation planning. However, routine identification of many species can be difficult and time-consuming, often requiring highly specialized knowledge, and therefore represents a limiting factor in biodiversity assessments and ecological studies [1-3]. In addition to this, the identification of larval stages or fragments of organisms using conventional morphological methods constitutes an impossible task for many taxa [4-6].

In this context, the use of DNA sequences represents a promising and effective tool for fast and accurate species identification [7-9]. Animal mitochondrial DNA exhibits several characteristics that makes it attractive for molecular taxonomy, namely the generally high substitution rates, the almost exclusively maternal inheritance, and the lack of recombination [10,11]. Moreover, because of uniparental inheritance and haploidy, mtDNA has a four-fold smaller effective population size compared to nuclear DNA, leading to faster lineage sorting [12]. A 650 base pair fragment of the 5' end of the mitochondrial cytochrome *c *oxidase I (COI) gene was proposed as global standard, the so-called "barcode region" for animals [7,13]. This barcode approach has been successfully applied in various vertebrate and invertebrate taxa for species delimitation and identification [14-19]. Subsets of the standard COI barcode have been shown to be effective for species-level identification in specimens whose DNA is degraded [20,21]. Nevertheless, the exclusive use of mitochondrial gene fragments is not without risks. The concept of DNA barcoding relies on low levels of mtDNA variation within species in combination with clear genetic differentiation between species, the so-called barcoding gap. Various studies found high levels of overlap in intra- and interspecific genetic distances for some selected taxa [22,23]. DNA barcoding can also overestimate the number of species when nuclear mitochondrial pseudogenes (numts) are coamplified [24-27]. Introgression events and/or incomplete lineage sorting can cause trans-specific polymorphisms in mitochondrial DNA, contorting the mitochondrial variability of studied organisms [28]. Such events have been demonstrated for various arthropod taxa, for example insects [29-33] or spiders [34,35]. Heteroplasmy events can also confuse the identification system also [36], but are rare [37]. Finally, maternally inherited endosymbionts such as the α-proteobacteriae *Wolbachia *or *Rickettsia *may cause linkage disequilibrium with mtDNA, resulting in a homogenization of mtDNA haplotypes [38-40].

All these problems show that standardised and complementing nuclear markers are necessary if a provisional species, uncovered using COI barcodes, is to be considered as species. In this context, nuclear ribosomal genes may represent potential supplementary markers for species identification. Nuclear ribosomal genes are generally considered to be highly conserved, but are actually composed of a mixture of conserved and variable regions that are organized in clusters that contain hundreds of copies per haploid genome. In metazoan taxa, these tandem rDNA units are highly uniform within a species [41-44], but differ between closely related species [e.g. [45-49]]. Until now, there have only been a few studies using nuclear rDNA sequences for DNA taxonomy: complete small ribosomal subunit DNA (18S rDNA) sequences were used to identify invertebrate taxa [1,5], while the variable D1-D2 or D3-D5 regions of the large ribosomal subunit DNA (28S rDNA) were found to be suitable markers for various fungi [50,51], arthropods [2,52,53] freshwater meiobenthic communities [54], and a broad range of metazoan taxa [55]. The main limitation to these approaches lies in the length of the analysed sequences (usually >>1000 base pairs (bp)), preventing a simple amplification of degraded DNA (e.g. from collection specimens in museums) and, most important, efficient use in large-scale biodiversity studies [56]. Nevertheless, it should be also noted that various potential problems can be associated with the use of ribosomal genes, for example intragenomic variations among rRNA gene copies. As far as we know, very few cases of intragenomic variations have been observed for Metazoa until now [57-63]. Multiple variants of the 18S rRNA gene were found in a dinoflagellate [64], a platyhelminth [65], and the Lake sturgeon *Acipenser fulvescens *[66,67].

While core elements of the eukaryotic ribosomal RNA genes are considered to be essential for ribosome functions that evolve slowly and evenly [68,69], the so-called divergence or expansion segments show a high variability in primary sequence and length between even closely related species as a consequence of DNA slippage-like processes [70-73]. In most cases, expansion segments have highly conserved flanking sites [68,69,74]. Although the exact functions remain elusive, various studies of eukaryotic ribosomes provide some clues about the functional aspects of expansion segments in rRNAs [75-77], including intersubunit bridges and scaffolds allowing proteins to bind to ribosomes [78]. In addition, some of their structural features seem to be important for the stability of rRNA [75,79,80].

Following these considerations, we analysed and compared the usefulness of nuclear ribosomal expansion segments and COI barcodes for the molecular identification of Central European carabid beetles. The Carabidae are among the largest and most diverse insect families, with no less than an estimated 40,000 described species that inhabit all terrestrial habitat types from the sub-arctic to wet tropical regions [81,82]. This diversity and wide distribution, along with the predominance of these beetles in a large variety of habitats, has resulted in a considerable interest in many aspects of their biology, including systematics, phylogeny, biogeography, ecology and evolution [83-87]. Ground beetles show different levels of habitat selectivity, ranging from generalists to specialists, and therefore carabid assemblages can be used as highly valuable bioindicators for characterizing disturbances in various habitats such as forests, meadows or fens [88]. Due to the continuous and intensive study of ground beetles in Central Europe, their taxonomic classification is well-established. In Central Europe, more than 750 species are known [89]. Nevertheless, the identification of many species and especially of larval stages can be very difficult as a consequence of high morphological variability within species and due to the existence of sibling species.

Our study examined the effectiveness and suitability of one mitochondrial (COI) and three nuclear markers, the expansion segments V4 and V7 of the 18S rDNA and the D3 expansion segment of the 28S rDNA as molecular identification tools for 75 selected ground beetle species out of 26 genera from Central Europe. We compared intra- and interspecific divergences using Kimura 2-parameter (K2P) distances and uncorrected *p*-distances between all analysed COI sequences and *p*-distances for all rDNA gene fragments of many closely related species, e.g. *Agonum emarginatum/viduum*, *Clivina collaris/fossor*, or *Harpalus affinis/rubripes*. Furthermore, we analysed the discrimination capacity of the used marker systems within two well-known pairs of sibling species, *Bembidion lampros/properans *[90-94] and *Pterostichus nigrita*/*rhaeticus *[95-98].

## Results

We examined 344 specimens representing 75 species and 28 genera of Central European ground beetles. The mitochondrial COI region and all three selected nuclear regions were successfully amplified and sequenced in all cases, confirming the universality of the selected primers for ground beetles. While the majority of the analysed beetles had been collected 1-2 years ago and were preserved in 96% ethanol, it was also possible to generate full length sequences with tissue samples of pinned specimens up to 12 years old. There was no indication of numt amplification for the COI dataset. Most importantly, we found no intragenomic or intraspecific variations within the analysed nuclear rDNA markers.

### The COI dataset

**Figure 1:**
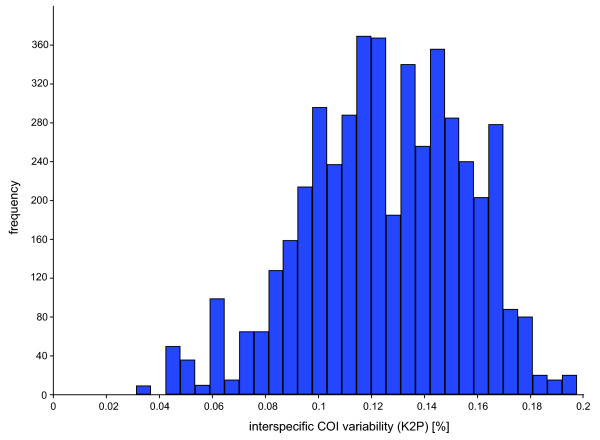
**Interspecific K2P divergences of the COI barcode fragment**. See Methods for more details.

**Figure 2:**
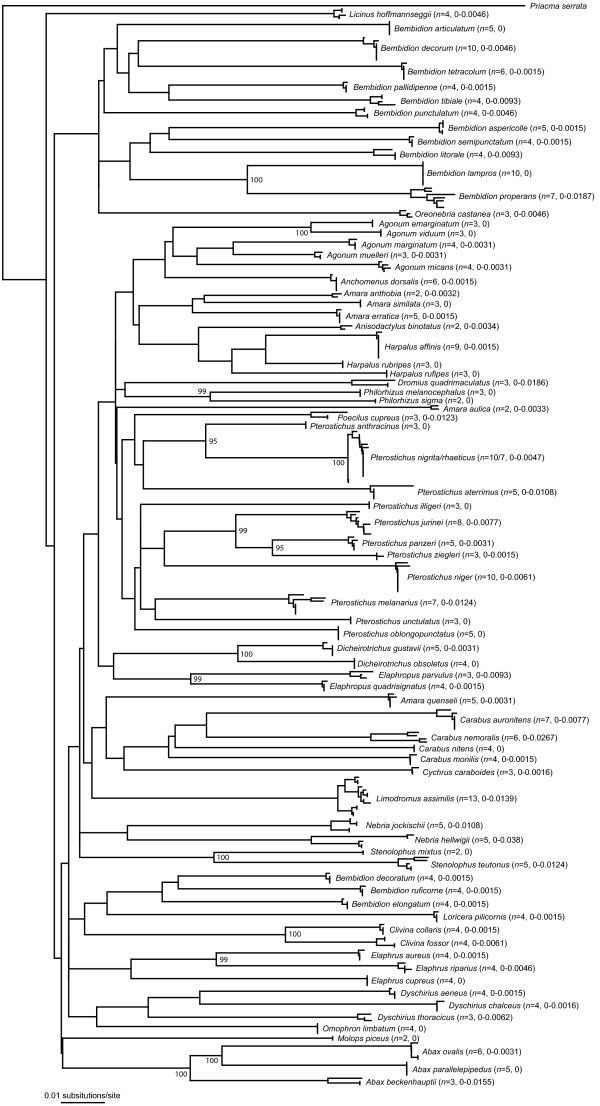
**Neighbor-joining tree of COI sequence divergences (K2P) in 75 ground beetle species from Central Europe**. Numbers next to internal branches are bootstrap values, which are only given if they have a value of 95 or more. Numbers in brackets indicate the number of analysed specimens (left) and intraspecific Kimura 2-parameter distance ranges (right).

**Figure 3:**
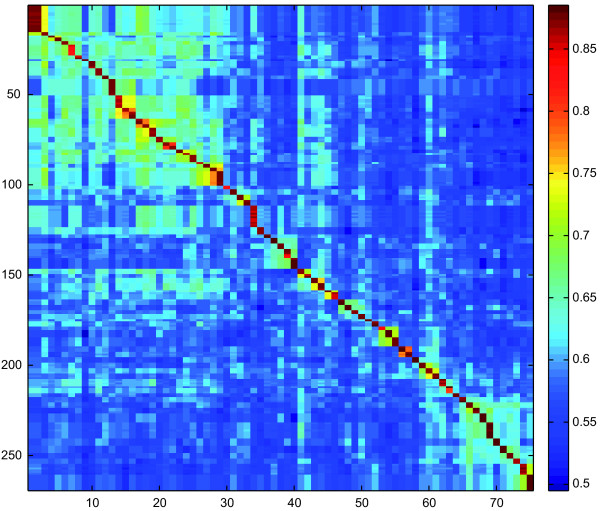
**Indicator vector correlations of the COI data set (Klee diagram)**. The false-color representation depicts correlations among 269 COI test sequences (y-axis) and 75 species-level indicator vectors (x-axis). In total, 263 (97.7%) of the test sequences showed highest correlation with their respective species indicator vector; exceptions were mis-assignments of *Pterostichus nigrita *and *Pterostichus rhaeticus*, which share predominant identical COI haplotypes.

All COI sequences were heavily AT biased, with an average A+T-content of 67.6%. The average interspecific K2P distance was 12.6% (*p*-distance: 11.5%), while the lowest distances were observed between *Agonum emarginatum *and *Agonum viduum *(K2P distances: 3.14%, *p*-distances: 3.06%) (Figure 1). Intraspecific distances ranged up to 3.8% for the analysed *Nebria hellwigii *specimens (*p*-distances: up to 3.7%), while specimens of *Carabus nemoralis *were characterized by K2P distances ranging up to 2.7% (*p*-distances: 2.6%) (Figure 2). However, both species revealed two distinct clades without intermediate haplotypes. In contrast to this, it was not possible to discriminate *Pterostichus nigrita *from *Pterostichus rhaeticus *using COI sequences. Both species shared various identical haplotypes, and K2P distances ranged from 0 up to 0.5% (*p*-distances: 0 to 0.5%) for both species (Figure 2, Table 1). A Klee diagram revealed correlation values > 0.75 for six species pairs, *Agonum emarginatum/viduum*, *Agonum marginatum*/*muelleri*, *Clivina collaris*/*fossor*, *Harpalus affinis*/*rubripes*, *Pterostichus nigrita*/*rhaeticus*, and *Pterostichus panzeri*/*ziegleri*, indicating a high similarity of the uncorrected COI sequences among these species pairs (Figure 3).

**Table 1:** Interspecific nucleotide divergences of all four markers, given in cases in which at least one of the analysed markers did not resolve the species pair

**Taxa pairs**	**28S: D3**	**18S: V4**	**18S: V7**	**28S: D3 +18S: V4+V7**	**COI**
*Agonum emarginatu/viduum*	identical sequences	0.0026/1	0.0025/1	0.002/2	0.0314
*Agonum marginatum/micans*	0.005/1	identical sequences	0.0025/1	0.0021/2	0.0793 - 0.0828
*Agonum marginatum/muelleri*	0.005/1	identical sequences	0.0025/1	0.0021/2	0.0506 - 0.0507
*Agonum micans/muelleri*	0.005/1	identical sequences	identical sequences	0.001/1	0.0809 - 0.0861
*Amara anthobia/erratica*	0.005/1	0.0028/1	identical sequences	0.0021/2	0.0677 - 0.0713
*Amara anthobia/similata*	identical sequences	0.0084/3	0.0274/11	0.0146/14	0.0713 - 0.0714
*Anisodactylus binotatus/Harpalus rubripes*	0.0302/6	identical sequences	0.005/2	0.0084/8	0.0723 - 0.0744
*Bembidion decorum/tetracolum*	identical sequences	0.0048/2	0.0145/6	0.0079/8	0.1245 - 0.1283
*Clivina collaris/fossor*	identical sequences	identical sequences	identical sequences	identical sequences	0.0446 - 0.0503
*Dyschirius aeneus/thoracicus*	0.0313/6	identical sequences	0.0119/5	0.0109/11	0.109 - 0.1126
*Elaphropus parvulus/quadrisignatus*	identical sequences	identical sequences	0.0075/3	0.003/3	0.0739 - 0.079
*Harpalus affinis/rubripes*	identical sequences	0.0028/1	0.0025/1	0.0021/2	0.0458
*Harpalus affinis/rufipes*	0.02/4	identical sequences	0.0025/1	0.0052/5	0.0706 - 0.0723
*Nebria hellwigii*	identical sequences	identical sequences	0.0024/1	0.001/1	0 - 0.038
*Pterostichus jurinei/ziegleri*	0.042/9	identical sequences	0.0125/5	0.0143/14	0.0573 - 0.064
*Pterostichus nigrita/rhaeticus*	identical sequences	identical sequences	identical sequences	identical sequences	identical sequences
*Pterostichus panzeri/jurinei*	0.0467/10	identical sequences	0.0125/5	0.0153/15	0.0572 - 0.0657
*Pterostichus panzeri/ziegleri*	0.014/3	identical sequences	identical sequences	0.0031/3	0.0442 - 0.0475
*Stenolophus mixtus/teutonus*	identical sequences	0.0111/4	0.005/2	0.0062/6	0.0865 - 0.0886

For COI sequences, values indicate the range of K2P divergences. For the nuclear markers, values indicate *p*-distances (left) and the number of base substitutions (right).

### The rDNA datasets

**Figure 4:**
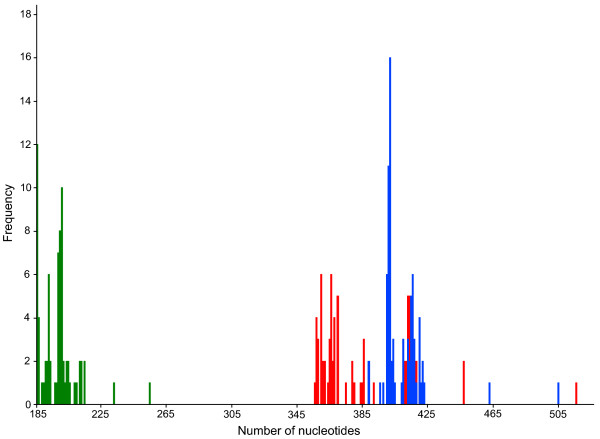
**Histogram of fragment length frequencies of the analysed V4 (red), V7 (blue) and D3 (green) sequences**. Fragment lengths from 185 to 254 bp for the D3 marker, 355 to 515 bp for the V4 marker, and 388 to 504 bp for the V7 marker.

**Figure 5:**
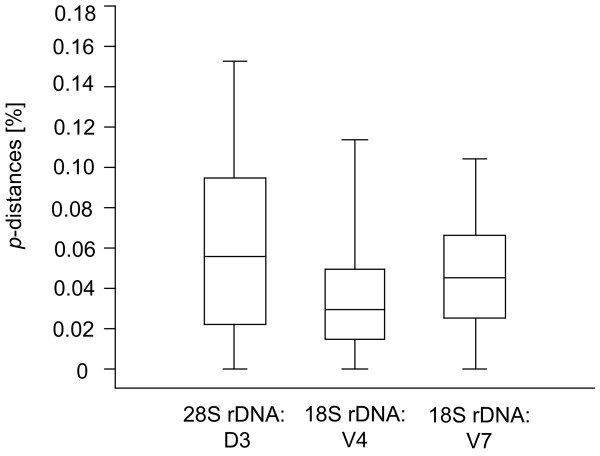
**Boxplot distribution of the interspecific *p*-distances for the D3, V4 and V7 gene fragments**. See Methods for methods of distance calculation and boxplot representations.

**Figure 6:**
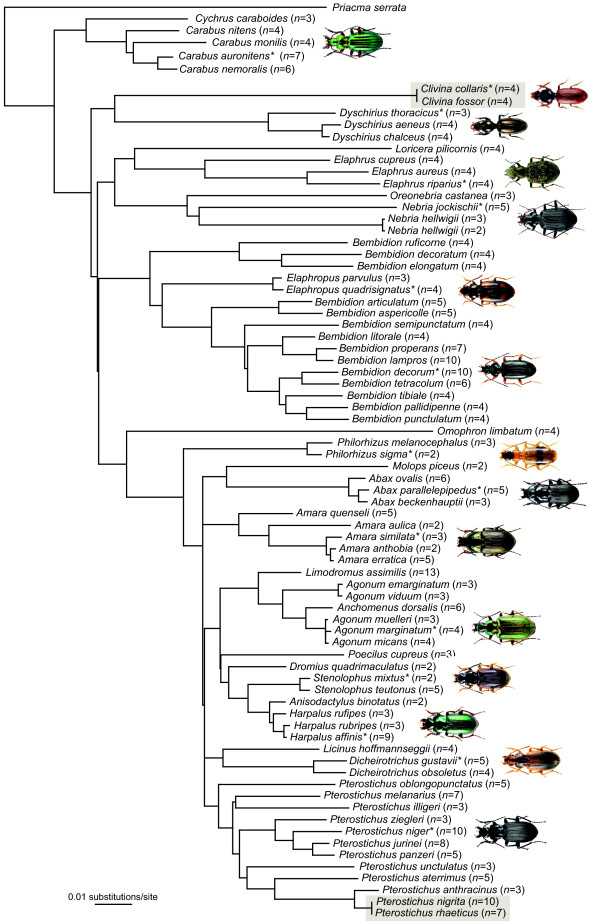
**Neighbor-joining tree of the concatenated D3, V4 and V7 expansion fragment datasets for 75 carabid species**. Values in brackets indicate the number of analysed specimens that were grouped together. Species with identical sequences are marked with grey boxes. Asterisks indicate representative ground beetles of genera with at least two analysed species for which illustrations are provided. All images were obtained from http://www.eurocarabidae.de.

Fragment lengths ranged from 185 (various species) to 254 bp (*Molops piceus*) with an average length of 198 bp for the D3 marker, 355 (*Dromius quadrimaculatus*) to 515 bp (*Omophron limbatum*) for the V4 marker (average length: 384 bp), and 388 (both studied *Philorhizus *species) to 504 bp (*Omophron limbatum*) for the V7 marker (average length: 414 bp) (Figure 4). Average *p*-distances between species within genera were 6.0% for the D3, 3.7% for the V4 and 4.6% for the V7 expansion fragment (Figure 5). Our study revealed that single base changes (substitutions, insertions or deletions) for all three analysed markers unambiguously separate closely related species, e.g. *Amara anthobia *and *Amara erratica *(D3), *Agonum viduum *and *Agonum marginatum *(V4), and *Harpalus affinis *and *Harpalus rubripes *(V7) (Table 1). Nevertheless, in some cases various species showed identical sequences and therefore no resolution, anticipating a successful species discrimination, e.g. *Elaphropus parvulus *and *Elaphropus quadrisignatus *for the D3 (Additional file 1), *Agonum marginatum*, *Agonum micans *and *Agonum muelleri *for the V4 (Additional file 2), and *Amara anthobia *and *Amara erratica *for the V7 marker (Additional file 3). All taxa without resolution are summarized in Table 1. In the case of both *Clivina *species and the sibling species *Pterostichus nigrita*/*rhaeticus*, no substitutions were observed in the studied nuclear sequences at all. Otherwise, it was possible to discriminate two different V7 sequences for *Nebria hellwigii*, differing in one base. Furthermore, both sequences correlated with the two distinct COI haplotype clusters (see above), while the two other nuclear markers showed no variation. Summarizing the results, it was possible to discriminate 61 (81%) species using the D3 marker, 57 (76%) using the V4 marker, and 65 (87%) using the V7 marker. The combined analyses of all three nuclear markers provided resolution for 71 (95%) species (Figure 6).

## Discussion

The issue of choosing thresholds for species delineation is a primary concern for molecular taxonomy [99], particularly when intraspecific variation can be shown to be greater than interspecific variation. In the case of COI, a species identification threshold has been suggested that amounts to ten times the arithmetic mean of intraspecific distances [100], which is usually low (less than 1%) and rarely more than 2% across a broad range of taxa [7,13,20,100-104]. However, this "10-fold rule" has been questioned in subsequent studies [105-107], because it has no strong biological background and undoubtedly cannot become a universal, invariable criterion to species delineation across all taxa [24,25,107]. Another approach comprises the analysis of mtDNA branching times using a general mixed Yule-coalescent (GMYC) model estimates the species boundary by identifying independently evolving lineages as a transition from coalescent to speciation branching patterns on a phylogenetic tree [108]. First case studies reveal the potential of this approach [108-111], but former bottleneck events or selective sweeps can become problematic in reconstructing the coalescence of mtDNA lineages and therefore for species delineation. It should be also kept in mind that such methods are also sensitive to introgression and incomplete lineage sorting, and cannot be used analyzing a high number of specimens.

Based on our data, lowest interspecific COI distances were observed between *Agonum emarginatum *and *Agonum viduum *(K2P distances: 3.14%, *p*-distances: 3.06%). With one exception (*Nebria hellwigii*), all intraspecific distances were below these values. Our results confirm the high potential of COI barcodes for species identification of even closely related carabid species although it was not possible to discriminate the two species of the *Pterostichus nigrita*/*rhaeticus *species complex (Figure 2, Figure 3, Table 1). Nevertheless, it is important to study additional specimens of the already analysed species, preferable from different locations, as well as missing species have to be analysed to gain more specific insights in the intra- and interspecific COI variability of ground beetles.

For molecular species identification, the use of rDNA is not a new approach [1,2,5,50-55]. However, published studies rely on the analysis of long rDNA fragments [2,50-55] or complete rDNA genes [1,5], currently preventing a routine use in large-scale biodiversity studies. As a consequence, our study was focused on analysing the usefulness of three short expansion segments from two different rRNA genes (18S and 28S) as supplementary molecular markers to the COI barcode region for ground beetles. In contrast to COI, the species identification threshold for all analysed rDNA marker had an amount of one base substitution, insertion or deletion. The individual identification success using the rDNA marker was limited: The D3 marker was able to discriminate 61 (81%) species, the V4 marker 57 (76%), and the V7 marker allowed an unambiguous identification of 65 (87%) species. However, the combination of all three nuclear markers provided resolution for 71 (95%) species. Only two species pairs, *Clivina collaris*/*fossor *and *Pterostichus nigrita*/*rhaeticus*, were not discriminated (Figure 6, Table 1). Summarizing all results, our data showed that COI represented the most successful molecular marker for species determination (73 = 97%) for the studied ground beetle species, closely followed by the combination of all three nuclear rDNA markers (71 = 95%).

The analysed nuclear ribosomal expansion segments show some important aspects that are quite useful in molecular species identification. Firstly, they show a significant species level genetic variability and divergence for most species when used in combination; the exclusive use of a single segment will not discriminate all analysed species. Secondly, all analysed fragments have appropriately short sequence lengths in comparison to the COI fragment, facilitating easy amplification and sequencing. Finally, highly conserved flanking sites allow the generation of primers useable for a broad range of taxa. However, it must be emphasized that the power of rDNA sequences for identifying species is limited when sister species pairs have a very recent origin, as suggested for *Clivina collaris *and *Clivina fossor*. In such cases, the analysis of COI sequences represents a more advantageous and effective approach, because substitution rates of mitochondrial genes are, in general, higher than those of nuclear rRNA genes. Nevertheless, the quality of other expansion segments to discriminate species, especially closely related ones, should be also tested in further studies.

Our analysed data revealed some important insights into the genetic variability of nuclear genes and mitochondrial genes of ground beetles that are discussed in more detail.

### The sibling species pair *Bembidion lampros*/*properans *- molecular data confirm two distinct species

Although overall morphological differences between both species are small, for example characteristic frontal furrows, the structure of the elytral striae and shape of the pronotum [98,102,112], both *Bembidion *species can be clearly distinguished by all molecular markers used. Interspecific K2P distances for COI ranged from 9 to 9.4% (*p*-distances: 8.4-8.8%), while the number of sequence substitutions ranged from three (V4) to four (V7, D3) for the analysed nuclear markers. For all four markers, sequence divergence between both species lay considerably above any of the thresholds suggested in the literature or our own study, indicating that *Bembidion lampros *and *Bembidion properans *are in fact two distinct species.

### The sibling species *Pterostichus nigrita*/*rhaeticus *- speciation in progress?

**Figure 7:**
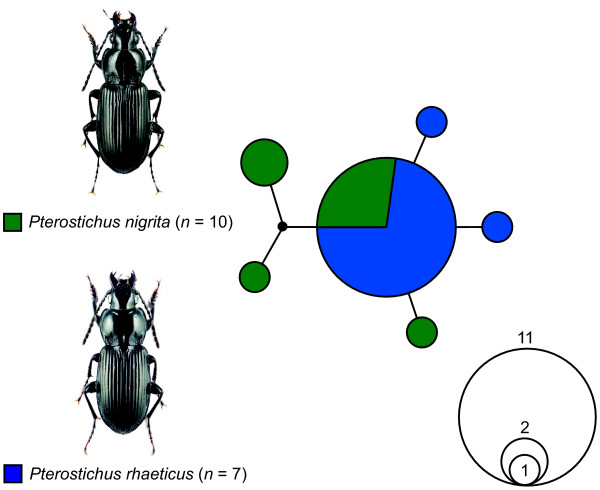
**Statistical parsimony network showing the mutational relationships among the analysed mitochondrial COI haplotypes of *Pterostichus nigrita *(green) and *Pterostichus rhaeticus *(blue)**. Each line in the network represents a single mutational change; small black dots indicate missing haplotypes. The numbers of analysed specimens (*n*) are listed; the diameter of the circles is proportional to the number of haplotypes sampled (see Open circles with numbers). Images were obtained from http://www.eurocarabidae.de.

Various ecological and crossbreeding studies give evidence of two long ignored but distinct species, *Pterostichus nigrita *and *Pterostichus rhaeticus *[95-98,113]. Overall morphological differences between both species are very subtle and hardly noticeable. Males may be identified by the shape of the inflated endophallus including intermediate stages, while females can be identified by the form of the eighth abdominal sternite [96-98]. Accordingly, there were no sequence variations within the three nuclear markers and the COI sequence divergence (K2P distances: 0 to 0.01%, *p*-distances: 0 to 0.01%) was very low. For COI, both species shared identical haplotypes, and there was no evidence for any differentiation among both species (Figure 7). It was not possible to consistently discriminate between both species using any of the analysed molecular markers in this study. However, when species pairs have very recent origins or hybridize, the use of DNA sequences for species identification is very limited: after the initial "split", new sister species will share alleles and mutations in slowly evolving genes [114]. Beside this, morphological distinctiveness may evolve much faster than the studied popular "standard" genes. Further specimens from various locations and different markers should be tested to understand the population structure of this species complex in more detail. Molecular markers with a higher resolution on population level, for example microsatellites or SNPs rather than single locus coding genes alone can give more insights into genetic variability and gene flow through migration and dispersal [10,11].

### *Clivina collaris*/*fossor *- recently evolved distinct species?

The ground beetle *Clivina collaris *represents the sister species of *Clivina fossor *and has, in the past, often been regarded as a variety of the former [112]. However, *Clivina collaris *is somewhat smaller and flatter than *Clivina fossor*, and various other morphological traits (the shape of the elytra, genital morphology, details of the inner armature and different ecological preferences) clearly support the existence of two closely related but distinct species [89,115]. Kimura 2-parameter distances of the COI sequences ranged from 4.5 to 5.3% (*p*-distance: 4.4-5.1%), while all three nuclear datasets revealed no sequence variation, indicating a possible recent separation of both species.

### *Nebria hellwigii *- more than one species?

The distribution of *Nebria hellwigii *is restricted to the alpine and high alpine regions of the Eastern Alps [89]. Based on genital morphology, a few subspecies are discussed [89]. Our data revealed two distinct COI haplotype lineages without intermediates and K2P distances of 3.8% (*p*-distance: 3.7%) that are higher than the lowest interspecific distances (K2P distances: 3.14%, *p*-distances: 3.06%, see above). However, both lineages were also identified by the nuclear V7 marker (Figure 6), giving evidence for the existence of a sibling species pair with sympatric distribution. Additional specimens have to be studied to evaluate these first results more in detail.

### *Carabus nemoralis *- indication of introgressive hybridization events and/or incomplete lineage sorting

A European wide-temperate and highly eurytopic species, *Carabus nemoralis *is widely distributed throughout Central and Northern Europe [89]. Beside significant shape variations between different populations [116,117], population genetic analyses using microsatellites revealed a complex genetic differentiation [118,119]. For this species, our COI data revealed two distinct haplotype lines with K2P distances of 2.7% (*p*-distances: 2.6%), without intermediates. However, all nuclear markers show no variation. Although we cannot exclude a linkage disequilibrium caused by inherited endosymbionts as well as heteroplasmy at the moment, introgressive hybridization events with other closely related *Carabus *species or incomplete lineage sorting represent the most plausible explanation for the observed high level of mitochondrial genetic differentiation. Both phenomena are well-known from other *Carabus *species and closely related genera [29-31,120]. In this case, the only use of COI sequences for molecular taxonomy using a threshold of 2% or below (see above) will clearly overestimate species diversity.

## Conclusions

Based on the analyses of our various datasets, we have come to the conclusion that nuclear ribosomal expansion segments can constitute a valuable and efficient supplement for classical DNA barcoding studies based on mitochondrial COI sequences. While the individual identification success using the rDNA marker was limited, the combination of all three nuclear markers provided resolution for 71 (95%) of the studied 75 ground beetle species. Using COI, 73 species (97%) of the studied ground beetle species were accurately identified. Our study confirms the high potential of DNA sequence data for successful species identification of even closely related ground beetle species.

## Methods

### DNA extraction, amplification and sequencing

For our study, a total number of 344 specimens belonging to 75 different species out of 26 carabid genera were analysed (Additional file 4). All beetles were identified by one of the authors of this article (KH). The characterization of intraspecific variation, introgression or other phenomena as well as the detection of cryptic species cannot be accomplished with only one individual per species; therefore at least two specimens per species and, if possible, specimens from different locations were analysed. The number of studied specimens per species ranged from a minimum of two to a maximum of 13, with four individuals per species on average (Additional file 5).

Genomic DNA samples were prepared from fresh beetles, beetles preserved in 96% ethanol or pinned museum specimens. Total genomic DNA was extracted from dissected legs of specimens or complete specimens using the QIAmp^© ^Tissue Kit (Qiagen GmbH, Hilden), following the manufacturers extraction protocol. Specimens are deposited in the collection of the Zoologisches Forschungsmuseum Alexander Koenig (ZFMK), Bonn, Germany. In addition, all analysed DNA extracts are deposited in the DNA bank of the ZFMK as part of the DNA Bank Network (http://www.dnabank-network.org).

In total, 344 sequences were newly generated for this study for all analysed gene fragments. GenBank accession numbers, tissue voucher depositories and collection site are listed in Additional file 4. Three sequences of one outgroup taxon (the cupedid beetle *Priacma serrata*, COI: EU839762, 18S rDNA: EU797411, 28S rDNA: EU797380) were obtained from GenBank. All 1356 amplification reactions were carried out on a Thermal Cycler GeneAmp^© ^PCR System 2700/2720 (Applied Biosystems, Darmstadt) in 20 μl volume, containing 4 μl Q-Solution, 2 μl 10 × Qiagen PCR buffer, 2 μl dNTPs (2 mmol/μl), 0.1 μl of each primer (both 50 pmol/μl), 1 μl of DNA template with an amount between 2 to 150 ng/μl, 0.2 μl Qiagen Taq polymerase (5 U/μl), and filled up to 20 μl with sterile H_2_O.

The PCR temperature profile for the mitochondrial COI fragment (approx. 650 bp) using the primers LCO1480 and HCO2198 [121] consisted of an initial denaturation at 94°C (5 min), followed by 38 cycles at 94°C (denaturation, 45 s), 48°C (annealing, 45 s), 72°C (extension, 80 s), and a final extension at 72°C (7 min). Approximately 200 bp of the D3 region was amplified with the forward primer CD3F and reverse primer CD3R, using a PCR protocol of 94°C for 5 min (initial denaturation), 32 cycles with 94°C denaturation for 45 s, 52°C annealing for 45 s, and 72°C extension for 80 s, followed by a final 72°C extension for 7 min. A 360-510 bp region of the V4 gene fragment was amplified with the primer pair CV4F and CV4R. The PCR temperature protocol was 94°C for 5 min (initial denaturation), 32 cycles with 94°C denaturation for 45 s, 66°C annealing for 45 s, and 72°C extension for 2 min, followed by a final 72°C extension for 8 min. Finally, a 400-500 base pair region of the V7 region of the 18S rDNA was amplified using the forward primer CV7F and either the reverse primer CV7R, with the following PCR conditions: 94°C for 5 min (initial denaturation), 32 cycles with 94°C denaturation for 45 s, 68°C annealing for 45 s, and 72°C extension for 2 min, followed by 72°C extension for 8 min. All primers used in amplification and sequencing for all four gene fragments as well as all used PCR temperature profiles are provided in Additional file 6.

Negative and positive controls were included with each round of reactions. Two μl of amplified product were verified for size conformity by electrophoresis in a 1% agarose gel with ethidium bromide using commercial DNA size standards, while the remaining PCR product was purified with the QIAquick^© ^PCR Purification Kit (Qiagen GmbH, Hilden). Purified PCR products were cycle sequenced and sequenced in both directions at a contract sequencing facility (Macrogen, Seoul, Korea) on an ABI3730 XL automatic DNA sequencer, using the same primers as used in PCR. Trace files revealed no potential ambiguities indicated by multiple peaks in the sequences. Double stranded sequences were assembled with the SeqMan™ II program (DNASTAR, Inc., Konstanz, Germany). BLAST searches were performed to confirm the identity of all new sequences [122]. All aligned COI sequences were translated to amino acid sequences to check for nuclear mitochondrial pseudogenes (numts) using BioEdit 7.0.9.0 [123].

### Data analysis

All sequences of each marker were aligned using MUSCLE version 3.6 [124] with default settings, generating four individual datasets. To analyse the intra- and interspecific genetic variability of species, uncorrected pairwise distances (*p*-distances) and Kimura 2-parameter (K2P) distances for the COI sequences, and *p*-distances for all rDNA marker were obtained using PAUP*4.0b10 [125]. Interspecific K2P distances of the COI data set were plotted as histogram (Figure 1), while *p*-distances of all rDNA data sets were visualized using box-and-whiskers-plots (Figure 5) [126], which represent the overall shape of the dataset. Boxes indicate mean, 25th and 75th percentile, while whiskers show 10th and 90th percentile, respectively. In contrast to protein-coding genes, the presence of multiple indels in alignments of ribosomal expansion regions makes accurate homology assessments across distantly related taxa difficult or even impossible. To accommodate this problem, all nuclear marker sequences of genera with at least two analysed species were aligned on genus level independent from all other taxa. Subsequently, sequence divergence calculations were carried out using PAUP*4.0b10 (Figure 5). Apart from this, the frequencies of the different lengths of the rDNA expansion fragment lengths were plotted as histogram (Figure 4). All boxplots and histograms were calculated using PAST version 1.94b [127]. We also generated a Klee diagram (Figure 3) based on indicator vector correlations for analyzing and displaying affinities of COI haplotypes [128,129]. Using this method, uncorrected COI haplotype sequences were transformed into digital indicator vectors using M = 1 sequence/species, generating unique representations of each sequence in the chosen vector space [128,129]. A false-color map depicts correlations among the remaining sequences and the species indicator vectors. The succession of species for this approach is provided in Additional file 7.

Neighbor-joining cluster analyses [130] were employed for graphical representation of patterns of nucleotide divergences among the individual specimens of the COI dataset (Figure 2), for each single rDNA dataset (D3: Additional file 1; V4: Additional file 2; V7: Additional file 3), and for a combined dataset including all three nuclear markers (Figure 6), using PAUP*4.0b10. The neighbor-joining analysis of the COI data set was based on K2P distances. Due to the fact that expansion segments cannot be aligned unambiguously in many cases, only *p*-distances were used for all four rDNA marker topologies. Bootstrap support values were obtained by re-sampling and analyzing 10,000 replicates for the COI data set. All alignments can be obtained from the first author upon request. In addition, a statistical parsimony network was constructed for the COI data sets of *Pterostichus nigrita *and *Pterostichus rhaeticus *with TCS 1.21 [131], using default settings (Figure 7).

It should be kept in mind that a deep phylogenetic signal is not of central importance for molecular (alpha) taxonomy, as it has been pointed out by various authors [132-134]. Instead of this, DNA barcoding and molecular taxonomy focus on species delineation and identification.

## Competing interests

The authors declare that they have no competing interests.

## Authors' contribution

MJR designed and coordinated the study, carried out the molecular analyses and drafted the manuscript. JJA, MKP and JWW contributed to the interpretation of the molecular data. KH provided most specimens and identified all analysed ground beetles. MYS helped with the generation of the Klee diagram. JWW initiated and oversaw the project. All authors contributed to the writing of the manuscript and approved the final manuscript.

## Supplementary Material

Additional file 1 **Neighbor-joining tree of D3 expansion fragments for 75 carabid species**. Numbers in brackets indicate the number of analysed specimens. Species with identical sequences are marked with grey boxes. Click here for file

Additional file 2 **Neighbor-joining tree of V4 expansion fragments for 75 carabid species**. Numbers in brackets indicate the number of analysed specimens. Species with identical sequences are marked with grey boxes. Click here for file

Additional file 3 **Neighbor-joining tree of V7 expansion fragments for 75 carabid species**. Numbers in brackets indicate the number of analysed specimens. Species with identical sequences are marked with grey boxes. Click here for file

Additional file 4 **Species identification, location of collection, and GenBank accession numbers of the analysed Carabidae**. Country codes: GER (Germany): NRW (Nordrhein-Westfalen), NS (Niedersachsen), SH (Schleswig-Holstein), RP (Rheinland-Pfalz), S (Sachsen), SA (Sachsen-Anhalt); AU (Austria): K (Kärnten), SM (Steiermark), and TI (Tirol). Click here for file

Additional file 5 **Histogram of studied specimens per species**. See Additional file 4 for more detailed information. Click here for file

Additional file 6 **Primers and PCR protocols used in this study**. Click here for file

Additional file 7 **Order of species used for the Klee diagram of indicator vector correlations of COI sequences**. The analysis is based on a Neighbor-joining (NJ) analysis using *p*-distances. Click here for file
