# Supplementary material for: Molecular species identification of Central European ground beetles (Coleoptera: Carabidae) using nuclear rDNA expansion segments and DNA barcodes
Source: Front Zool. 2010 Sep 13;7:26. doi: 10.1186/1742-9994-7-26 (PMC2945340; doi:10.1186/1742-9994-7-26)
Supplement: Additional file 4 — Species identification, location of collection, and GenBank accession numbers of the analysed Carabidae. Country codes: GER (Germany): NRW (Nordrhein-Westfalen), NS (Niedersachsen), SH (Schleswig-Holstein), RP (Rheinland-Pfalz), S (Sachsen), SA (Sachsen-Anhalt); AU (Austria): K (Kärnten), SM (Steiermark), and TI (Tirol). [file 1742-9994-7-26-S4.DOC]

| **Tribus** | **Genus** | **ZFMK voucher code** | **Locality** | **CO1** | **28S rDNA:**  **D3** | **18S rDNA:**  **V4** | **18S rDNA:**  **V7** |
| --- | --- | --- | --- | --- | --- | --- | --- |
| Omophronini | *Omophron limbatum* (Fabricius, 1776) | ZFMK_COL_2008_47 | Bienen, NRW, GER | GU347265 | GU347609 | GU347953 | GU348297 |
|  | *Omophron limbatum* (Fabricius, 1776) | ZFMK_COL_2009_258 | Bockholt, NRW, GER | GU347266 | GU347610 | GU347954 | GU348298 |
|  | *Omophron limbatum* (Fabricius, 1776) | ZFMK_COL_2009_259 | Bockholt, NRW, GER | GU347267 | GU347611 | GU347955 | GU348299 |
|  | *Omophron limbatum* (Fabricius, 1776) | ZFMK_COL_2009_260 | Schmedehausen, NRW, GER | GU347268 | GU347612 | GU347956 | GU348300 |
| Carabini | *Carabus auronitens* (Fabricius, 1792) | ZFMK_COL_2008_51 | Bad Iburg, NS, GER | GU347140 | GU347484 | GU347828 | GU348172 |
|  | *Carabus auronitens* (Fabricius, 1792) | ZFMK_COL_2008_52 | Bad Iburg, NS, GER | GU347142 | GU347486 | GU347830 | GU348174 |
|  | *Carabus auronitens* (Fabricius, 1792) | ZFMK_COL_2008_53 | Bad Iburg, NS, GER | GU347143 | GU347487 | GU347831 | GU348175 |
|  | *Carabus auronitens* (Fabricius, 1792) | ZFMK_COL_2008_54 | Bad Iburg, NS, GER | GU347144 | GU347488 | GU347832 | GU348176 |
|  | *Carabus auronitens* (Fabricius, 1792) | ZFMK_COL_2008_55 | Bad Iburg, NS, GER | GU347145 | GU347489 | GU347833 | GU348177 |
|  | *Carabus auronitens* (Fabricius, 1792) | ZFMK_COL_2008_56 | Bad Iburg, NS, GER | GU347146 | GU347490 | GU347834 | GU348178 |
|  | *Carabus auronitens* (Fabricius, 1792) | ZFMK_COL_2009_544 | Totes Gebirge, SM, AU | GU347141 | GU347485 | GU347829 | GU348173 |
|  | *Carabus monilis* Fabricius, 1792 | ZFMK_COL_2009_417 | Leverkusen, NRW, GER | GU347147 | GU347491 | GU347835 | GU348179 |
|  | *Carabus monilis* Fabricius, 1792 | ZFMK_COL_2009_418 | Leverkusen, NRW, GER | GU347148 | GU347492 | GU347836 | GU348180 |
|  | *Carabus monilis* Fabricius, 1792 | ZFMK_COL_2009_419 | Leverkusen, NRW, GER | GU347149 | GU347493 | GU347837 | GU348181 |
|  | *Carabus monilis* Fabricius, 1792 | ZFMK_COL_2009_420 | Leverkusen, NRW, GER | GU347150 | GU347494 | GU347838 | GU348182 |
|  | *Carabus nemoralis* Müller, 1764 | ZFMK_COL_2008_241 | Waltrop, NRW, GER | GU347151 | GU347495 | GU347839 | GU348183 |
|  | *Carabus nemoralis* Müller, 1764 | ZFMK_COL_2008_243 | Waltrop, NRW, GER | GU347153 | GU347497 | GU347841 | GU348185 |
|  | *Carabus nemoralis* Müller, 1764 | ZFMK_COL_2008_397 | Fehmarn, SH, GER | GU347154 | GU347498 | GU347842 | GU348186 |
|  | *Carabus nemoralis* Müller, 1764 | ZFMK_COL_2008_398 | Fehmarn, SH, GER | GU347152 | GU347496 | GU347840 | GU348184 |
|  | *Carabus nemoralis* Müller, 1764 | ZFMK_COL_2008_399 | Fehmarn, SH, GER | GU347155 | GU347499 | GU347843 | GU348187 |
|  | *Carabus nemoralis* Müller, 1764 | ZFMK_COL_2009_673 | Waltrop, NRW, GER | GU347156 | GU347500 | GU347844 | GU348188 |
|  | *Carabus nitens* Linne, 1758 | ZFMK_COL_2008_72 | Haltern-Borkenberge, NRW, GER | GU347157 | GU347501 | GU347845 | GU348189 |
|  | *Carabus nitens* Linne, 1758 | ZFMK_COL_2008_74 | Haltern-Borkenberge, NRW, GER | GU347158 | GU347502 | GU347846 | GU348190 |
|  | *Carabus nitens* Linne, 1758 | ZFMK_COL_2008_77 | Haltern-Borkenberge, NRW, GER | GU347159 | GU347503 | GU347847 | GU348191 |
|  | *Carabus nitens* Linne, 1758 | ZFMK_COL_2009_683 | Haltern-Borkenberge, NRW, GER | GU347160 | GU347504 | GU347848 | GU348192 |
| Cychrini | *Cychrus caraboides* (Linne, 1758) | ZFMK_COL_2009_537 | Zillertaler Alpen, TI, AU | GU347169 | GU347513 | GU347857 | GU348201 |
|  | *Cychrus caraboides* (Linne, 1758) | ZFMK_COL_2009_538 | Zillertaler Alpen, TI, AU | GU347171 | GU347515 | GU347859 | GU348203 |
|  | *Cychrus caraboides* (Linne, 1758) | ZFMK_COL_2009_539 | Zillertaler Alpen, TI, AU | GU347170 | GU347514 | GU347858 | GU348202 |
| Nebriini | *Nebria hellwigii* (Panzer, 1803) | ZFMK_COL_2009_577 | Totes Gebirge, SM, AU | GU347260 | GU347604 | GU347948 | GU348292 |
|  | *Nebria hellwigii* (Panzer, 1803) | ZFMK_COL_2009_582 | Zillertaler Alpen, TI, AU | GU347261 | GU347605 | GU347949 | GU348293 |
|  | *Nebria hellwigii* (Panzer, 1803) | ZFMK_COL_2009_583 | Zillertaler Alpen, TI, AU | GU347262 | GU347606 | GU347950 | GU348294 |
|  | *Nebria hellwigii* (Panzer, 1803) | ZFMK_COL_2009_584 | Totes Gebirge, SM, AU | GU347263 | GU347607 | GU347951 | GU348295 |
|  | *Nebria hellwigii* (Panzer, 1803) | ZFMK_COL_2009_585 | Zillertaler Alpen, TI, AU | GU347264 | GU347608 | GU347952 | GU348296 |
|  | *Nebria jockischii* Sturm, 1815 | ZFMK_COL_2009_558 | Zillertaler Alpen, TI, AU | GU347255 | GU347599 | GU347943 | GU348287 |
|  | *Nebria jockischii* Sturm, 1815 | ZFMK_COL_2009_559 | Zillertaler Alpen, TI, AU | GU347257 | GU347601 | GU347945 | GU348289 |
|  | *Nebria jockischii* Sturm, 1815 | ZFMK_COL_2009_560 | Zillertaler Alpen, TI, AU | GU347258 | GU347602 | GU347946 | GU348290 |
|  | *Nebria jockischii* Sturm, 1815 | ZFMK_COL_2009_561 | Zillertaler Alpen, TI, AU | GU347259 | GU347603 | GU347947 | GU348291f |
|  | *Nebria jockischii* Sturm, 1815 | ZFMK_COL_2009_562 | Zillertaler Alpen, TI, AU | GU347256 | GU347600 | GU347944 | GU348288 |
|  | *Oreonebria castanea* (Bonelli, 1810) | ZFMK_COL_2009_565 | Zillertaler Alpen, TI, AU | GU347252 | GU347596 | GU347940 | GU348284 |
|  | *Oreonebria castanea* (Bonelli, 1810) | ZFMK_COL_2009_566 | Zillertaler Alpen, TI, AU | GU347254 | GU347598 | GU347942 | GU348286 |
|  | *Oreonebria castanea* (Bonelli, 1810) | ZFMK_COL_2009_570 | Zillertaler Alpen, TI, AU | GU347253 | GU347597 | GU347941 | GU348285 |
| Elaphrini | *Elaphrus aureus* Müller, 1821 | ZFMK_COL_2009_202 | Schmedehausen, NRW, GER | GU347202 | GU347546 | GU347890 | GU348234 |
|  | *Elaphrus aureus* Müller, 1821 | ZFMK_COL_2009_203 | Schmedehausen, NRW, GER | GU347203 | GU347547 | GU347891 | GU348235 |
|  | *Elaphrus aureus* Müller, 1821 | ZFMK_COL_2009_204 | Schmedehausen, NRW, GER | GU347204 | GU347548 | GU347892 | GU348236 |
|  | *Elaphrus aureus* Müller, 1821 | ZFMK_COL_2009_205 | Schmedehausen, NRW, GER | GU347205 | GU347549 | GU347893 | GU348237 |
|  | *Elaphrus cupreus* (Linne, 1758) | ZFMK_COL_2008_352 | Waltrop, NRW, GER | GU347206 | GU347550 | GU347894 | GU348238 |
|  | *Elaphrus cupreus* (Linne, 1758) | ZFMK_COL_2008_369 | Fehmarn, SH, GER | GU347207 | GU347551 | GU347895 | GU348239 |
|  | *Elaphrus cupreus* (Linne, 1758) | ZFMK_COL_2008_370 | Fehmarn, SH, GER | GU347208 | GU347552 | GU347896 | GU348240 |
|  | *Elaphrus cupreus* (Linne, 1758) | ZFMK_COL_2008_371 | Fehmarn, SH, GER | GU347209 | GU347553 | GU347897 | GU348241 |
|  | *Elaphrus riparius* (Linne, 1758) | ZFMK_COL_2008_48 | Bienen, NRW, GER | GU347210 | GU347554 | GU347898 | GU348242 |
|  | *Elaphrus riparius* (Linne, 1758) | ZFMK_COL_2009_206 | Schmedehausen, NRW, GER | GU347211 | GU347555 | GU347899 | GU348243 |
|  | *Elaphrus riparius* (Linne, 1758) | ZFMK_COL_2009_207 | Schmedehausen, NRW, GER | GU347212 | GU347556 | GU347900 | GU348244 |
|  | *Elaphrus riparius* (Linne, 1758) | ZFMK_COL_2009_208 | Schmedehausen, NRW, GER | GU347213 | GU347557 | GU347901 | GU348245 |
| Loricerini | *Loricera pilicornis* (Fabricius, 1775) | ZFMK_COL_2008_499 | Waltrop, NRW, GER | GU347246 | GU347590 | GU347934 | GU348278 |
|  | *Loricera pilicornis* (Fabricius, 1775) | ZFMK_COL_2008_500 | Waltrop, NRW, GER | GU347248 | GU347592 | GU347936 | GU348280 |
|  | *Loricera pilicornis* (Fabricius, 1775) | ZFMK_COL_2008_501 | Waltrop, NRW, GER | GU347249 | GU347593 | GU347937 | GU348281 |
|  | *Loricera pilicornis* (Fabricius, 1775) | ZFMK_COL_2009_575 | Billerbeck, NRW, GER | GU347247 | GU347591 | GU347935 | GU348279 |
| Scaritini | *Clivina collaris* (Herbst, 1784) | ZFMK_COL_2009_151 | Schmedehausen, NRW, GER | GU347161 | GU347505 | GU347849 | GU348193 |
|  | *Clivina collaris* (Herbst, 1784) | ZFMK_COL_2009_152 | Schmedehausen, NRW, GER | GU347162 | GU347506 | GU347850 | GU348194 |
|  | *Clivina collaris* (Herbst, 1784) | ZFMK_COL_2009_153 | Schmedehausen, NRW, GER | GU347163 | GU347507 | GU347851 | GU348195 |
|  | *Clivina collaris* (Herbst, 1784) | ZFMK_COL_2009_154 | Schmedehausen, NRW, GER | GU347164 | GU347508 | GU347852 | GU348196 |
|  | *Clivina fossor* (Linne, 1758) | ZFMK_COL_2009_159 | Schmedehausen, NRW, GER | GU347165 | GU347509 | GU347853 | GU348197 |
|  | *Clivina fossor* (Linne, 1758) | ZFMK_COL_2009_160 | Fehmarn, SH, GER | GU347166 | GU347510 | GU347854 | GU348198 |
|  | *Clivina fossor* (Linne, 1758) | ZFMK_COL_2009_163 | Castrop-Rauxel, NRW, GER | GU347167 | GU347511 | GU347855 | GU348199 |
|  | *Clivina fossor* (Linne, 1758) | ZFMK_COL_2009_164 | Castrop-Rauxel, NRW, GER | GU347168 | GU347512 | GU347856 | GU348200 |
|  | *Dyschirius aeneus* (Dejean, 1825) | ZFMK_COL_2009_183 | Schmedehausen, NRW, GER | GU347184 | GU347528 | GU347872 | GU348216 |
|  | *Dyschirius aeneus* (Dejean, 1825) | ZFMK_COL_2009_184 | Schmedehausen, NRW, GER | GU347185 | GU347529 | GU347873 | GU348217 |
|  | *Dyschirius aeneus* (Dejean, 1825) | ZFMK_COL_2009_185 | Schmedehausen, NRW, GER | GU347186 | GU347530 | GU347874 | GU348218 |
|  | *Dyschirius aeneus* (Dejean, 1825) | ZFMK_COL_2009_186 | Schmedehausen, NRW, GER | GU347187 | GU347531 | GU347875 | GU348219 |
|  | *Dyschirius chalceus* Erichson, 1837 | ZFMK_COL_2009_429 | Halle an der Saale, SA, GER | GU347191 | GU347535 | GU347879 | GU348223 |
|  | *Dyschirius chalceus* Erichson, 1837 | ZFMK_COL_2009_430 | Halle an der Saale, SA, GER | GU347188 | GU347532 | GU347876 | GU348220 |
|  | *Dyschirius chalceus* Erichson, 1837 | ZFMK_COL_2009_431 | Halle an der Saale, SA, GER | GU347189 | GU347533 | GU347877 | GU348221 |
|  | *Dyschirius chalceus* Erichson, 1837 | ZFMK_COL_2009_432 | Halle an der Saale, SA, GER | GU347190 | GU347534 | GU347878 | GU348222 |
|  | *Dyschirius thoracicus* (Rossi, 1790) | ZFMK_COL_2008_41 | Bienen, NRW, GER | GU347192 | GU347536 | GU347880 | GU348224 |
|  | *Dyschirius thoracicus* (Rossi, 1790) | ZFMK_COL_2009_196 | Fehmarn, SH, GER | GU347193 | GU347537 | GU347881 | GU348225 |
|  | *Dyschirius thoracicus* (Rossi, 1790) | ZFMK_COL_2009_197 | Fehmarn, SH, GER | GU347194 | GU347538 | GU347882 | GU348226 |
| Bembidiini | *Elaphropus parvulus* (Dejean, 1831) | ZFMK_COL_2009_198 | Castrop-Rauxel, NRW, GER | GU347195 | GU347539 | GU347883 | GU348227 |
|  | *Elaphropus parvulus* (Dejean, 1831) | ZFMK_COL_2009_199 | Castrop-Rauxel, NRW, GER | GU347196 | GU347540 | GU347884 | GU348228 |
|  | *Elaphropus parvulus* (Dejean, 1831) | ZFMK_COL_2009_200 | Castrop-Rauxel, NRW, GER | GU347197 | GU347541 | GU347885 | GU348229 |
|  | *Elaphropus quadrisignatus* (Duftschmid, 1812) | ZFMK_COL_2009_437 | Maria Elend, K, AU | GU347201 | GU347545 | GU347889 | GU348233 |
|  | *Elaphropus quadrisignatus* (Duftschmid, 1812) | ZFMK_COL_2009_438 | Maria Elend, K, AU | GU347198 | GU347542 | GU347886 | GU348230 |
|  | *Elaphropus quadrisignatus* (Duftschmid, 1812) | ZFMK_COL_2009_439 | Feistritz, K, AU | GU347199 | GU347543 | GU347887 | GU348231 |
|  | *Elaphropus quadrisignatus* (Duftschmid, 1812) | ZFMK_COL_2009_440 | Feistritz, K, AU | GU347200 | GU347544 | GU347888 | GU348232 |
|  | *Bembidion articulatum* (Panzer, 1796) | ZFMK_COL_2008_44 | Bienen, NRW, GER | GU347065 | GU347409 | GU347753 | GU348097 |
|  | *Bembidion articulatum* (Panzer, 1796) | ZFMK_COL_2008_99 | Waltrop, NRW, GER | GU347068 | GU347412 | GU347756 | GU348100 |
|  | *Bembidion articulatum* (Panzer, 1796) | ZFMK_COL_2008_119 | Waltrop, NRW, GER | GU347069 | GU347413 | GU347757 | GU348101 |
|  | *Bembidion articulatum* (Panzer, 1796) | ZFMK_COL_2008_159 | Waltrop, NRW, GER | GU347067 | GU347411 | GU347755 | GU348099 |
|  | *Bembidion articulatum* (Panzer, 1796) | ZFMK_COL_2009_38 | Schmedehausen, NRW, GER | GU347066 | GU347410 | GU347754 | GU348098 |
|  | *Bembidion aspericolle* (Panzer, 1796) | ZFMK_COL_2008_23 | Teutschenthal, SA, GER | GU347070 | GU347414 | GU347758 | GU348102 |
|  | *Bembidion aspericolle* (Panzer, 1796) | ZFMK_COL_2008_24 | Teutschenthal, SA, GER | GU347072 | GU347416 | GU347760 | GU348104 |
|  | *Bembidion aspericolle* (Panzer, 1796) | ZFMK_COL_2008_25 | Teutschenthal, SA, GER | GU347073 | GU347417 | GU347761 | GU348105 |
|  | *Bembidion aspericolle* (Panzer, 1796) | ZFMK_COL_2008_26 | Teutschenthal, SA, GER | GU347074 | GU347418 | GU347762 | GU348106 |
|  | *Bembidion aspericolle* (Panzer, 1796) | ZFMK_COL_2008_27 | Teutschenthal, SA, GER | GU347071 | GU347415 | GU347759 | GU348103 |
|  | *Bembidion decoratum* (Duftschmid, 1812) | ZFMK_COL_2009_361 | Maria Elend, K, AU | GU347075 | GU347419 | GU347763 | GU348107 |
|  | *Bembidion decoratum* (Duftschmid, 1812) | ZFMK_COL_2009_362 | Maria Elend, K, AU | GU347076 | GU347420 | GU347764 | GU348108 |
|  | *Bembidion decoratum* (Duftschmid, 1812) | ZFMK_COL_2009_363 | Maria Elend, K, AU | GU347077 | GU347421 | GU347765 | GU348109 |
|  | *Bembidion decoratum* (Duftschmid, 1812) | ZFMK_COL_2009_364 | Maria Elend, K, AU | GU347078 | GU347422 | GU347766 | GU348110 |
|  | *Bembidion decorum* (Panzer, 1799) | ZFMK_COL_2008_12 | Neheim, NRW, GER | GU347079 | GU347423 | GU347767 | GU348111 |
|  | *Bembidion decorum* (Panzer, 1799) | ZFMK_COL_2008_13 | Neheim, NRW, GER | GU347081 | GU347425 | GU347769 | GU348113 |
|  | *Bembidion decorum* (Panzer, 1799) | ZFMK_COL_2008_14 | Neheim, NRW, GER | GU347082 | GU347426 | GU347770 | GU348114 |
|  | *Bembidion decorum* (Panzer, 1799) | ZFMK_COL_2008_16 | Bienen, NRW, GER | GU347083 | GU347427 | GU347771 | GU348115 |
|  | *Bembidion decorum* (Panzer, 1799) | ZFMK_COL_2008_42 | St. Augustin-Meindorf, NRW, GER | GU347080 | GU347424 | GU347768 | GU348112 |
|  | *Bembidion decorum* (Panzer, 1799) | ZFMK_COL_2009_360 | St. Augustin-Meindorf, NRW, GER | GU347085 | GU347429 | GU347773 | GU348117 |
|  | *Bembidion decorum* (Panzer, 1799) | ZFMK_COL_2009_400 | St. Augustin-Meindorf, NRW, GER | GU347084 | GU347428 | GU347772 | GU348116 |
|  | *Bembidion decorum* (Panzer, 1799) | ZFMK_COL_2009_498 | St. Augustin-Meindorf, NRW, GER | GU347086 | GU347430 | GU347774 | GU348118 |
|  | *Bembidion decorum* (Panzer, 1799) | ZFMK_COL_2009_499 | St. Augustin-Meindorf, NRW, GER | GU347087 | GU347431 | GU347775 | GU348119 |
|  | *Bembidion decorum* (Panzer, 1799) | ZFMK_COL_2009_500 | Bienen, NRW, GER | GU347088 | GU347432 | GU347776 | GU348120 |
|  | *Bembidion elongatum* Dejean, 1831 | ZFMK_COL_2009_40 | Schmedehausen, NRW, GER | GU347089 | GU347433 | GU347777 | GU348121 |
|  | *Bembidion elongatum* Dejean, 1831 | ZFMK_COL_2009_41 | Schmedehausen, NRW, GER | GU347090 | GU347434 | GU347778 | GU348122 |
|  | *Bembidion elongatum* Dejean, 1831 | ZFMK_COL_2009_42 | Schmedehausen, NRW, GER | GU347091 | GU347435 | GU347779 | GU348123 |
|  | *Bembidion elongatum* Dejean, 1831 | ZFMK_COL_2009_43 | Schmedehausen, NRW, GER | GU347092 | GU347436 | GU347780 | GU348124 |
|  | *Bembidion lampros* (Herbst, 1787) | ZFMK_COL_2008_122 | Borkum, NS, GER | GU347094 | GU347438 | GU347782 | GU348126 |
|  | *Bembidion lampros* (Herbst, 1787) | ZFMK_COL_2008_142 | Arnsberg, NRW, GER | GU347096 | GU347440 | GU347784 | GU348128 |
|  | *Bembidion lampros* (Herbst, 1787) | ZFMK_COL_2008_146 | Haltern-Lavesum, NRW, GER | GU347097 | GU347441 | GU347785 | GU348129 |
|  | *Bembidion lampros* (Herbst, 1787) | ZFMK_COL_2009_53 | Schmedehausen, NRW, GER | GU347101 | GU347445 | GU347789 | GU348133 |
|  | *Bembidion lampros* (Herbst, 1787) | ZFMK_COL_2009_54 | Schmedehausen, NRW, GER | GU347102 | GU347446 | GU347790 | GU348134 |
|  | *Bembidion lampros* (Herbst, 1787) | ZFMK_COL_2009_611 | Teutschenthal, SA, GER | GU347098 | GU347442 | GU347786 | GU348130 |
|  | *Bembidion lampros* (Herbst, 1787) | ZFMK_COL_2009_612 | Teutschenthal, SA, GER | GU347093 | GU347437 | GU347781 | GU348125 |
|  | *Bembidion lampros* (Herbst, 1787) | ZFMK_COL_2009_613 | Teutschenthal, SA, GER | GU347099 | GU347443 | GU347787 | GU348131 |
|  | *Bembidion lampros* (Herbst, 1787) | ZFMK_COL_2009_614 | Teutschenthal, SA, GER | GU347100 | GU347444 | GU347788 | GU348132 |
|  | *Bembidion lampros* (Herbst, 1787) | ZFMK_COL_2009_644 | Wyk, SH, GER | GU347095 | GU347439 | GU347783 | GU348127 |
|  | *Bembidion litorale* (Olivier, 1790) | ZFMK_COL_2009_59 | Bockholt, NRW, GER | GU347103 | GU347447 | GU347791 | GU348135 |
|  | *Bembidion litorale* (Olivier, 1790) | ZFMK_COL_2009_60 | Bockholt, NRW, GER | GU347105 | GU347449 | GU347793 | GU348137 |
|  | *Bembidion litorale* (Olivier, 1790) | ZFMK_COL_2009_61 | Bockholt, NRW, GER | GU347106 | GU347450 | GU347794 | GU348138 |
|  | *Bembidion litorale* (Olivier, 1790) | ZFMK_COL_2009_62 | Bockholt, NRW, GER | GU347104 | GU347448 | GU347792 | GU348136 |
|  | *Bembidion pallidipenne* (Illiger, 1802) | ZFMK_COL_2009_67 | Fehmarn, SH, GER | GU347107 | GU347451 | GU347795 | GU348139 |
|  | *Bembidion pallidipenne* (Illiger, 1802) | ZFMK_COL_2009_68 | Fehmarn, SH, GER | GU347108 | GU347452 | GU347796 | GU348140 |
|  | *Bembidion pallidipenne* (Illiger, 1802) | ZFMK_COL_2009_69 | Fehmarn, SH, GER | GU347109 | GU347453 | GU347797 | GU348141 |
|  | *Bembidion pallidipenne* (Illiger, 1802) | ZFMK_COL_2009_70 | Fehmarn, SH, GER | GU347110 | GU347454 | GU347798 | GU348142 |
|  | *Bembidion properans* (Stephens, 1828) | ZFMK_COL_2009_75 | Castrop-Rauxel, NRW, GER | GU347116 | GU347460 | GU347804 | GU348148 |
|  | *Bembidion properans* (Stephens, 1828) | ZFMK_COL_2009_76 | Castrop-Rauxel, NRW, GER | GU347117 | GU347461 | GU347805 | GU348149 |
|  | *Bembidion properans* (Stephens, 1828) | ZFMK_COL_2009_77 | Castrop-Rauxel, NRW, GER | GU347112 | GU347456 | GU347800 | GU348144 |
|  | *Bembidion properans* (Stephens, 1828) | ZFMK_COL_2009_615 | Teutschenthal, SA, GER | GU347111 | GU347455 | GU347799 | GU348143 |
|  | *Bembidion properans* (Stephens, 1828) | ZFMK_COL_2009_616 | Teutschenthal, SA, GER | GU347113 | GU347457 | GU347801 | GU348145 |
|  | *Bembidion properans* (Stephens, 1828) | ZFMK_COL_2009_617 | Teutschenthal, SA, GER | GU347114 | GU347458 | GU347802 | GU348146 |
|  | *Bembidion properans* (Stephens, 1828) | ZFMK_COL_2009_618 | Teutschenthal, SA, GER | GU347118 | GU347459 | GU347803 | GU348147 |
|  | *Bembidion punctulatum* Drapiez, 1820 | ZFMK_COL_2008_39 | Bienen, NRW, GER | GU347136 | GU347480 | GU347824 | GU348168 |
|  | *Bembidion punctulatum* Drapiez, 1820 | ZFMK_COL_2009_382 | Dessau, SA, GER | GU347139 | GU347483 | GU347827 | GU348171 |
|  | *Bembidion punctulatum* Drapiez, 1820 | ZFMK_COL_2009_383 | Dessau, SA, GER | GU347138 | GU347482 | GU347826 | GU348170 |
|  | *Bembidion punctulatum* Drapiez, 1820 | ZFMK_COL_2009_384 | Neheim, NRW, GER | GU347137 | GU347481 | GU347825 | GU348169 |
|  | *Bembidion ruficorne* Sturm, 1825 | ZFMK_COL_2009_391 | Feistritz, K, AU | GU347118 | GU347462 | GU347806 | GU348150 |
|  | *Bembidion ruficorne* Sturm, 1825 | ZFMK_COL_2009_392 | Feistritz, K, AU | GU347119 | GU347463 | GU347807 | GU348151 |
|  | *Bembidion ruficorne* Sturm, 1825 | ZFMK_COL_2009_393 | Feistritz, K, AU | GU347120 | GU347464 | GU347808 | GU348152 |
|  | *Bembidion ruficorne* Sturm, 1825 | ZFMK_COL_2009_394 | Feistritz, K, AU | GU347121 | GU347465 | GU347809 | GU348153 |
|  | *Bembidion semipunctatum*(Donovan, 1806) | ZFMK_COL_2008_43 | Bienen, NRW, GER | GU347123 | GU347467 | GU347811 | GU348155 |
|  | *Bembidion semipunctatum*(Donovan, 1806) | ZFMK_COL_2009_396 | Bienen, NRW, GER | GU347122 | GU347466 | GU347810 | GU348154 |
|  | *Bembidion semipunctatum*(Donovan, 1806) | ZFMK_COL_2009_397 | Bienen, NRW, GER | GU347124 | GU347468 | GU347812 | GU348156 |
|  | *Bembidion semipunctatum*(Donovan, 1806) | ZFMK_COL_2009_398 | Bienen, NRW, GER | GU347125 | GU347469 | GU347813 | GU348157 |
|  | *Bembidion tetracolum* Say, 1823 | ZFMK_COL_2009_90 | Schmedehausen, NRW, GER | GU347126 | GU347470 | GU347814 | GU348158 |
|  | *Bembidion tetracolum* Say, 1823 | ZFMK_COL_2009_91 | Schmedehausen, NRW, GER | GU347130 | GU347474 | GU347818 | GU348162 |
|  | *Bembidion tetracolum* Say, 1823 | ZFMK_COL_2009_92 | Schmedehausen, NRW, GER | GU347127 | GU347471 | GU347815 | GU348159 |
|  | *Bembidion tetracolum* Say, 1823 | ZFMK_COL_2009_93 | Schmedehausen, NRW, GER | GU347128 | GU347472 | GU347816 | GU348160 |
|  | *Bembidion tetracolum* Say, 1823 | ZFMK_COL_2009_94 | Schmedehausen, NRW, GER | GU347129 | GU347473 | GU347817 | GU348161 |
|  | *Bembidion tetracolum* Say, 1823 | ZFMK_COL_2009_601 | Remagen, RP, GER | GU347131 | GU347475 | GU347819 | GU348163 |
|  | *Bembidion tibiale* (Duftschmid, 1812) | ZFMK_COL_2009_404 | Nachrodt-Wiblingwerde, NRW,GER | GU347132 | GU347476 | GU347820 | GU348164 |
|  | *Bembidion tibiale* (Duftschmid, 1812) | ZFMK_COL_2009_405 | Nachrodt-Wiblingwerde, NRW,GER | GU347133 | GU347477 | GU347821 | GU348165 |
|  | *Bembidion tibiale* (Duftschmid, 1812) | ZFMK_COL_2009_406 | Zell-Mitterwinkel, K, AU | GU347134 | GU347478 | GU347822 | GU348166 |
|  | *Bembidion tibiale* (Duftschmid, 1812) | ZFMK_COL_2009_407 | Zell-Mitterwinkel, K, AU | GU347135 | GU347479 | GU347823 | GU348167 |
| Pterostichini | *Poecilus cupreus* (Linne, 1758) | ZFMK_COL_2008_46 | Bienen, NRW, GER | GU347274 | GU347618 | GU347962 | GU348306 |
|  | *Poecilus cupreus* (Linne, 1758) | ZFMK_COL_2008_57 | Waltrop, NRW, GER | GU347275 | GU347619 | GU347963 | GU348307 |
|  | *Poecilus cupreus* (Linne, 1758) | ZFMK_COL_2008_58 | Waltrop, NRW, GER | GU347276 | GU347620 | GU347964 | GU348308 |
|  | *Pterostichus anthracinus* (Illiger, 1798) | ZFMK_COL_2008_392 | Fehmarn, SH, GER | GU347277 | GU347621 | GU347965 | GU348309 |
|  | *Pterostichus anthracinus* (Illiger, 1798) | ZFMK_COL_2008_394 | Fehmarn, SH, GER | GU347278 | GU347622 | GU347966 | GU348310 |
|  | *Pterostichus anthracinus* (Illiger, 1798) | ZFMK_COL_2008_395 | Fehmarn, SH, GER | GU347279 | GU347623 | GU347967 | GU348311 |
|  | *Pterostichus aterrimus* (Herbst, 1784) | ZFMK_COL_2008_382 | Fehmarn, SH, GER | GU347280 | GU347624 | GU347968 | GU348312 |
|  | *Pterostichus aterrimus* (Herbst, 1784) | ZFMK_COL_2008_383 | Fehmarn, SH, GER | GU347281 | GU347625 | GU347969 | GU348313 |
|  | *Pterostichus aterrimus* (Herbst, 1784) | ZFMK_COL_2008_384 | Fehmarn, SH, GER | GU347282 | GU347626 | GU347970 | GU348314 |
|  | *Pterostichus aterrimus* (Herbst, 1784) | ZFMK_COL_2008_385 | Fehmarn, SH, GER | GU347283 | GU347627 | GU347971 | GU348315 |
|  | *Pterostichus aterrimus* (Herbst, 1784) | ZFMK_COL_2008_386 | Fehmarn, SH, GER | GU347284 | GU347628 | GU347972 | GU348316 |
|  | *Pterostichus illigeri* (Panzer, 1803) | ZFMK_COL_2009_465 | Gurktaler Alpen, K, AU | GU347285 | GU347629 | GU347973 | GU348317 |
|  | *Pterostichus illigeri* (Panzer, 1803) | ZFMK_COL_2009_466 | Gurktaler Alpen, K, AU | GU347286 | GU347630 | GU347974 | GU348318 |
|  | *Pterostichus illigeri* (Panzer, 1803) | ZFMK_COL_2009_467 | Gurktaler Alpen, K, AU | GU347287 | GU347631 | GU347975 | GU348319 |
|  | *Pterostichus jurinei* (Duftschmid, 1812) | ZFMK_COL_2009_469 | Gurktaler Alpen, K, AU | GU347293 | GU347637 | GU347981 | GU348325 |
|  | *Pterostichus jurinei* (Duftschmid, 1812) | ZFMK_COL_2009_470 | Gurktaler Alpen, K, AU | GU347294 | GU347638 | GU347982 | GU348326 |
|  | *Pterostichus jurinei* (Duftschmid, 1812) | ZFMK_COL_2009_471 | Gurktaler Alpen, K, AU | GU347295 | GU347639 | GU347983 | GU348327 |
|  | *Pterostichus jurinei* (Duftschmid, 1812) | ZFMK_COL_2009_536 | Zillertaler Alpen, TI, AU | GU347288 | GU347632 | GU347976 | GU348320 |
|  | *Pterostichus jurinei* (Duftschmid, 1812) | ZFMK_COL_2009_545 | Totes Gebirge, SM, AU | GU347290 | GU347634 | GU347978 | GU348322 |
|  | *Pterostichus jurinei* (Duftschmid, 1812) | ZFMK_COL_2009_550 | Zillertaler Alpen, TI, AU | GU347291 | GU347635 | GU347979 | GU348323 |
|  | *Pterostichus jurinei* (Duftschmid, 1812) | ZFMK_COL_2009_551 | Zillertaler Alpen, TI, AU | GU347292 | GU347636 | GU347980 | GU348324 |
|  | *Pterostichus jurinei* (Duftschmid, 1812) | ZFMK_COL_2009_552 | Zillertaler Alpen, TI, AU | GU347289 | GU347633 | GU347977 | GU348321 |
|  | *Pterostichus melanarius* (Illiger, 1798) | ZFMK_COL_2008_380 | Fehmarn, SH, GER | GU347296 | GU347640 | GU347984 | GU348328 |
|  | *Pterostichus melanarius* (Illiger, 1798) | ZFMK_COL_2008_406 | Fehmarn, SH, GER | GU347297 | GU347641 | GU347985 | GU348329 |
|  | *Pterostichus melanarius* (Illiger, 1798) | ZFMK_COL_2008_415 | Fehmarn, SH, GER | GU347299 | GU347643 | GU347987 | GU348331 |
|  | *Pterostichus melanarius* (Illiger, 1798) | ZFMK_COL_2008_431 | Fehmarn, SH, GER | GU347302 | GU347646 | GU347990 | GU348334 |
|  | *Pterostichus melanarius* (Illiger, 1798) | ZFMK_COL_2008_432 | Fehmarn, SH, GER | GU347300 | GU347644 | GU347988 | GU348332 |
|  | *Pterostichus melanarius* (Illiger, 1798) | ZFMK_COL_2008_433 | Fehmarn, SH, GER | GU347301 | GU347645 | GU347989 | GU348333 |
|  | *Pterostichus melanarius* (Illiger, 1798) | ZFMK_COL_2008_434 | Fehmarn, SH, GER | GU347298 | GU347642 | GU347986 | GU348330 |
|  | *Pterostichus niger* (Schaller, 1783) | ZFMK_COL_2008_248 | Waltrop, NRW, GER | GU347303 | GU347647 | GU347991 | GU348335 |
|  | *Pterostichus niger* (Schaller, 1783) | ZFMK_COL_2008_249 | Waltrop, NRW, GER | GU347305 | GU347649 | GU347993 | GU348337 |
|  | *Pterostichus niger* (Schaller, 1783) | ZFMK_COL_2008_253 | Waltrop, NRW, GER | GU347306 | GU347650 | GU347994 | GU348338 |
|  | *Pterostichus niger* (Schaller, 1783) | ZFMK_COL_2008_387 | Fehmarn, SH, GER | GU347307 | GU347651 | GU347995 | GU348339 |
|  | *Pterostichus niger* (Schaller, 1783) | ZFMK_COL_2008_388 | Fehmarn, SH, GER | GU347308 | GU347652 | GU347996 | GU348340 |
|  | *Pterostichus niger* (Schaller, 1783) | ZFMK_COL_2008_423 | Fehmarn, SH, GER | GU347309 | GU347653 | GU347997 | GU348341 |
|  | *Pterostichus niger* (Schaller, 1783) | ZFMK_COL_2008_424 | Fehmarn, SH, GER | GU347310 | GU347654 | GU347998 | GU348342 |
|  | *Pterostichus niger* (Schaller, 1783) | ZFMK_COL_2008_430 | Fehmarn, SH, GER | GU347311 | GU347655 | GU347999 | GU348343 |
|  | *Pterostichus niger* (Schaller, 1783) | ZFMK_COL_2008_485 | Waltrop, NRW, GER | GU347304 | GU347648 | GU347992 | GU348336 |
|  | *Pterostichus niger* (Schaller, 1783) | ZFMK_COL_2009_497 | Bad Münstereifel, NRW, GER | GU347312 | GU347656 | GU348000 | GU348344 |
|  | *Pterostichus nigrita* (Paykull, 1790) | ZFMK_COL_2009_590 | Waltrop, NRW, GER | GU347313 | GU347657 | GU348001 | GU348345 |
|  | *Pterostichus nigrita* (Paykull, 1790) | ZFMK_COL_2009_591 | Waltrop, NRW, GER | GU347315 | GU347659 | GU348003 | GU348347 |
|  | *Pterostichus nigrita* (Paykull, 1790) | ZFMK_COL_2009_592 | Waltrop, NRW, GER | GU347316 | GU347660 | GU348004 | GU348348 |
|  | *Pterostichus nigrita* (Paykull, 1790) | ZFMK_COL_2009_593 | Waltrop, NRW, GER | GU347317 | GU347661 | GU348005 | GU348349 |
|  | *Pterostichus nigrita* (Paykull, 1790) | ZFMK_COL_2009_594 | Waltrop, NRW, GER | GU347318 | GU347662 | GU348006 | GU348350 |
|  | *Pterostichus nigrita* (Paykull, 1790) | ZFMK_COL_2009_595 | Waltrop, NRW, GER | GU347319 | GU347663 | GU348007 | GU348351 |
|  | *Pterostichus nigrita* (Paykull, 1790) | ZFMK_COL_2009_596 | Waltrop, NRW, GER | GU347320 | GU347664 | GU348008 | GU348352 |
|  | *Pterostichus nigrita* (Paykull, 1790) | ZFMK_COL_2009_597 | Waltrop, NRW, GER | GU347321 | GU347665 | GU348009 | GU348353 |
|  | *Pterostichus nigrita* (Paykull, 1790) | ZFMK_COL_2009_598 | Waltrop, NRW, GER | GU347322 | GU347666 | GU348010 | GU348354 |
|  | *Pterostichus nigrita* (Paykull, 1790) | ZFMK_COL_2009_610 | Nachrodt-Wiblingwerde, NRW, GER | GU347314 | GU347658 | GU348002 | GU348346 |
|  | *Pterostichus oblongopunctatus* (Fabricius, 1787) | ZFMK_COL_2008_33 | Haltern-Borkenberge, NRW, GER | GU347323 | GU347667 | GU348011 | GU348355 |
|  | *Pterostichus oblongopunctatus* (Fabricius, 1787) | ZFMK_COL_2008_34 | Haltern-Borkenberge, NRW, GER | GU347324 | GU347668 | GU348012 | GU348356 |
|  | *Pterostichus oblongopunctatus* (Fabricius, 1787) | ZFMK_COL_2008_35 | Haltern-Borkenberge, NRW, GER | GU347325 | GU347669 | GU348013 | GU348357 |
|  | *Pterostichus oblongopunctatus* (Fabricius, 1787) | ZFMK_COL_2008_36 | Haltern-Borkenberge, NRW, GER | GU347326 | GU347670 | GU348014 | GU348358 |
|  | *Pterostichus oblongopunctatus* (Fabricius, 1787) | ZFMK_COL_2008_37 | Haltern-Borkenberge, NRW, GER | GU347327 | GU347671 | GU348015 | GU348359 |
|  | *Pterostichus panzeri* (Panzer, 1803) | ZFMK_COL_2009_587 | Totes Gebirge, SM, AU | GU347329 | GU347673 | GU348017 | GU348361 |
|  | *Pterostichus panzeri* (Panzer, 1803) | ZFMK_COL_2009_546 | Totes Gebirge, SM, AU | GU347328 | GU347672 | GU348016 | GU348360 |
|  | *Pterostichus panzeri* (Panzer, 1803) | ZFMK_COL_2009_547 | Totes Gebirge, SM, AU | GU347330 | GU347674 | GU348018 | GU348362 |
|  | *Pterostichus panzeri* (Panzer, 1803) | ZFMK_COL_2009_548 | Totes Gebirge, SM, AU | GU347331 | GU347675 | GU348019 | GU348363 |
|  | *Pterostichus panzeri* (Panzer, 1803) | ZFMK_COL_2009_549 | Totes Gebirge, SM, AU | GU347332 | GU347676 | GU348020 | GU348364 |
|  | *Pterostichus rhaeticus* Heer, 1837 | ZFMK_COL_2009_576 | Fehmarn, SH, GER | GU347333 | GU347677 | GU348021 | GU348365 |
|  | *Pterostichus rhaeticus* Heer, 1837 | ZFMK_COL_2009_658 | Willroth, RP, GER | GU347334 | GU347678 | GU348022 | GU348366 |
|  | *Pterostichus rhaeticus* Heer, 1837 | ZFMK_COL_2009_659 | Willroth, RP, GER | GU347335 | GU347679 | GU348023 | GU348367 |
|  | *Pterostichus rhaeticus* Heer, 1837 | ZFMK_COL_2009_660 | Willroth, RP, GER | GU347336 | GU347680 | GU348024 | GU348368 |
|  | *Pterostichus rhaeticus* Heer, 1837 | ZFMK_COL_2009_661 | Willroth, RP, GER | GU347337 | GU347681 | GU348025 | GU348369 |
|  | *Pterostichus rhaeticus* Heer, 1837 | ZFMK_COL_2009_662 | Willroth, RP, GER | GU347338 | GU347682 | GU348026 | GU348370 |
|  | *Pterostichus rhaeticus* Heer, 1837 | ZFMK_COL_2009_663 | Willroth, RP, GER | GU347339 | GU347683 | GU348027 | GU348371 |
|  | *Pterostichus unctulatus* (Duftschmid, 1812) | ZFMK_COL_2009_480 | Gurktaler Alpen, K, AU | GU347340 | GU347684 | GU348028 | GU348372 |
|  | *Pterostichus unctulatus* (Duftschmid, 1812) | ZFMK_COL_2009_481 | Gurktaler Alpen, K, AU | GU347342 | GU347686 | GU348029 | GU348373 |
|  | *Pterostichus unctulatus* (Duftschmid, 1812) | ZFMK_COL_2009_482 | Gurktaler Alpen, K, AU | GU347341 | GU347685 | GU348030 | GU348374 |
|  | *Pterostichus ziegleri* (Duftschmid, 1812) | ZFMK_COL_2009_484 | Hochobir, K, AU | GU347343 | GU347687 | GU348031 | GU34835 |
|  | *Pterostichus ziegleri* (Duftschmid, 1812) | ZFMK_COL_2009_485 | Hochobir, K, AU | GU347344 | GU347688 | GU348032 | GU348376 |
|  | *Pterostichus ziegleri* (Duftschmid, 1812) | ZFMK_COL_2009_486 | Hochobir, K, AU | GU347345 | GU347689 | GU348033 | GU348377 |
|  | *Molops piceus* (Panzer, 1793) | ZFMK_COL_2008_438 | Meinerzhagen, NRW, GER | GU347250 | GU347594 | GU347938 | GU348282 |
|  | *Molops piceus* (Panzer, 1793) | ZFMK_COL_2009_447 | Bad Münstereifel, NRW, GER | GU347251 | GU347595 | GU347939 | GU348283 |
|  | *Abax beckenhauptii* Schauberger, 1927 | ZFMK_COL_2009_312 | Hochobir, K, AU | GU347010 | GU347354 | GU347698 | GU348042 |
|  | *Abax beckenhauptii* Schauberger, 1927 | ZFMK_COL_2009_313 | Hochobir, K, AU | GU347011 | GU347355 | GU347699 | GU348043 |
|  | *Abax beckenhauptii* Schauberger, 1927 | ZFMK_COL_2009_629 | Feistritz, K, AU | GU347009 | GU347353 | GU347697 | GU348041 |
|  | *Abax ovalis* (Duftschmid, 1812) | ZFMK_COL_2008_439 | Lennestadt, NRW, GER | GU347012 | GU347356 | GU347700 | GU348044 |
|  | *Abax ovalis* (Duftschmid, 1812) | ZFMK_COL_2008_440 | Lennestadt, NRW, GER | GU347014 | GU347358 | GU347702 | GU348046 |
|  | *Abax ovalis* (Duftschmid, 1812) | ZFMK_COL_2008_442 | Drolshagen, NRW, GER | GU347015 | GU347359 | GU347703 | GU348047 |
|  | *Abax ovalis* (Duftschmid, 1812) | ZFMK_COL_2008_443 | Drolshagen, NRW, GER | GU347016 | GU347360 | GU347704 | GU348048 |
|  | *Abax ovalis* (Duftschmid, 1812) | ZFMK_COL_2008_444 | Drolshagen, NRW, GER | GU347017 | GU347361 | GU347705 | GU348049 |
|  | *Abax ovalis* (Duftschmid, 1812) | ZFMK_COL_2008_445 | Drolshagen, NRW, GER | GU347013 | GU347357 | GU347701 | GU348045 |
|  | *Abax parallelepipedus* (Piller & Mitterp., 1783) | ZFMK_COL_2009_314 | Waltrop, NRW, GER | GU347018 | GU347362 | GU347706 | GU348050 |
|  | *Abax parallelepipedus* (Piller & Mitterp., 1783) | ZFMK_COL_2009_315 | Waltrop, NRW, GER | GU347021 | GU347365 | GU347709 | GU348053 |
|  | *Abax parallelepipedus* (Piller & Mitterp., 1783) | ZFMK_COL_2009_316 | Waltrop, NRW, GER | GU347022 | GU347366 | GU347710 | GU348054 |
|  | *Abax parallelepipedus* (Piller & Mitterp., 1783) | ZFMK_COL_2009_599 | Bad Münstereifel, NRW, GER | GU347020 | GU347364 | GU347708 | GU348052 |
|  | *Abax parallelepipedus* (Piller & Mitterp., 1783) | ZFMK_COL_2009_573 | Rolandswerth, RP, GER | GU347019 | GU347363 | GU347707 | GU348051 |
| Platynini | *Limodromus assimilis* (Paykull, 1790) | ZFMK_COL_2008_277 | Blankenberg, NRW, GER | GU347233 | GU347577 | GU347921 | GU348265 |
|  | *Limodromus assimilis* (Paykull, 1790) | ZFMK_COL_2008_281 | Haltern-Lavesum, NRW, GER | GU347235 | GU347579 | GU347923 | GU348267 |
|  | *Limodromus assimilis* (Paykull, 1790) | ZFMK_COL_2008_295 | Blankenberg, NRW, GER | GU347236 | GU347580 | GU347924 | GU348268 |
|  | *Limodromus assimilis* (Paykull, 1790) | ZFMK_COL_2008_416 | Haltern-Borkenberge, NRW, GER | GU347237 | GU347581 | GU347925 | GU348269 |
|  | *Limodromus assimilis* (Paykull, 1790) | ZFMK_COL_2008_417 | Fehmarn, SH, GER | GU347238 | GU347582 | GU347926 | GU348270 |
|  | *Limodromus assimilis* (Paykull, 1790) | ZFMK_COL_2008_418 | Fehmarn, SH, GER | GU347239 | GU347583 | GU347927 | GU348271 |
|  | *Limodromus assimilis* (Paykull, 1790) | ZFMK_COL_2008_419 | Fehmarn, SH, GER | GU347240 | GU347584 | GU347928 | GU348272 |
|  | *Limodromus assimilis* (Paykull, 1790) | ZFMK_COL_2008_420 | Fehmarn, SH, GER | GU347241 | GU347585 | GU347929 | GU348273 |
|  | *Limodromus assimilis* (Paykull, 1790) | ZFMK_COL_2008_421 | Fehmarn, SH, GER | GU347242 | GU347586 | GU347930 | GU348274 |
|  | *Limodromus assimilis* (Paykull, 1790) | ZFMK_COL_2008_454 | Fehmarn, SH, GER | GU347243 | GU347587 | GU347931 | GU348275 |
|  | *Limodromus assimilis* (Paykull, 1790) | ZFMK_COL_2008_455 | Fehmarn, SH, GER | GU347244 | GU347588 | GU347932 | GU348276 |
|  | *Limodromus assimilis* (Paykull, 1790) | ZFMK_COL_2008_456 | Fehmarn, SH, GER | GU347234 | GU347578 | GU347922 | GU348266 |
|  | *Limodromus assimilis* (Paykull, 1790) | ZFMK_COL_2009_572 | Rolandswerth, RP, GER | GU347245 | GU347589 | GU347933 | GU348277 |
|  | *Anchomenus dorsalis* (Pontoppidan, 1763) | ZFMK_COL_2008_461 | Fehmarn, SH, GER | GU347060 | GU347404 | GU347748 | GU348092 |
|  | *Anchomenus dorsalis* (Pontoppidan, 1763) | ZFMK_COL_2008_462 | Fehmarn, SH, GER | GU347061 | GU347405 | GU347749 | GU348093 |
|  | *Anchomenus dorsalis* (Pontoppidan, 1763) | ZFMK_COL_2008_463 | Fehmarn, SH, GER | GU347062 | GU347406 | GU347750 | GU348094 |
|  | *Anchomenus dorsalis* (Pontoppidan, 1763) | ZFMK_COL_2008_466 | Fehmarn, SH, GER | GU347058 | GU347402 | GU347746 | GU348090 |
|  | *Anchomenus dorsalis* (Pontoppidan, 1763) | ZFMK_COL_2009_567 | Billerbeck, NRW, GER | GU347057 | GU347401 | GU347745 | GU348089 |
|  | *Anchomenus dorsalis* (Pontoppidan, 1763) | ZFMK_COL_2009_568 | Billerbeck, NRW, GER | GU347059 | GU347403 | GU347747 | GU348091 |
|  | *Agonum emarginatum* (Gyllenhal, 1827) | ZFMK_COL_2008_516 | Waltrop, NRW, GER | GU347023 | GU347367 | GU347711 | GU348055 |
|  | *Agonum emarginatum* (Gyllenhal, 1827) | ZFMK_COL_2008_517 | Waltrop, NRW, GER | GU347025 | GU347369 | GU347713 | GU348057 |
|  | *Agonum emarginatum* (Gyllenhal, 1827) | ZFMK_COL_2009_7 | Schmedehausen, NRW, GER | GU347024 | GU347368 | GU347712 | GU348056 |
|  | *Agonum marginatum* (Linne, 1758) | ZFMK_COL_2009_321 | Pouch, SA, GER | GU347026 | GU347370 | GU347714 | GU348058 |
|  | *Agonum marginatum* (Linne, 1758) | ZFMK_COL_2009_322 | Pouch, SA, GER | GU347028 | GU347372 | GU347716 | GU348060 |
|  | *Agonum marginatum* (Linne, 1758) | ZFMK_COL_2009_323 | Pouch, SA, GER | GU347029 | GU347373 | GU347717 | GU348061 |
|  | *Agonum marginatum* (Linne, 1758) | ZFMK_COL_2009_324 | Pouch, SA, GER | GU347027 | GU347371 | GU347715 | GU348059 |
|  | *Agonum micans* (Nicolai, 1822) | ZFMK_COL_2008_525 | Waltrop, NRW, GER | GU347030 | GU347374 | GU347718 | GU348062 |
|  | *Agonum micans* (Nicolai, 1822) | ZFMK_COL_2008_526 | Waltrop, NRW, GER | GU347031 | GU347375 | GU347719 | GU348063 |
|  | *Agonum micans* (Nicolai, 1822) | ZFMK_COL_2008_527 | Waltrop, NRW, GER | GU347032 | GU347376 | GU347720 | GU348064 |
|  | *Agonum micans* (Nicolai, 1822) | ZFMK_COL_2008_528 | Waltrop, NRW, GER | GU347033 | GU347377 | GU347721 | GU348065 |
|  | *Agonum muelleri* (Herbst, 1784) | ZFMK_COL_2009_501 | St. Augustin-Meindorf, NRW, GER | GU347034 | GU347378 | GU347722 | GU348066 |
|  | *Agonum muelleri* (Herbst, 1784) | ZFMK_COL_2009_502 | St. Augustin, NRW, GER | GU347035 | GU347379 | GU347723 | GU348067 |
|  | *Agonum muelleri* (Herbst, 1784) | ZFMK_COL_2009_503 | St. Augustin, NRW, GER | GU347036 | GU347380 | GU347724 | GU348068 |
|  | *Agonum viduum* (Panzer, 1797) | ZFMK_COL_2008_211 | Waltrop, NRW, GER | GU347037 | GU347381 | GU347725 | GU348069 |
|  | *Agonum viduum* (Panzer, 1797) | ZFMK_COL_2008_214 | Waltrop, NRW, GER | GU347038 | GU347382 | GU347726 | GU348070 |
|  | *Agonum viduum* (Panzer, 1797) | ZFMK_COL_2008_215 | Waltrop, NRW, GER | GU347039 | GU347383 | GU347727 | GU348071 |
| Zabrini | *Amara anthobia* A. & G.B. Villa, 1833 | ZFMK_COL_2008_85 | Waltrop, NRW, GER | GU347040 | GU347384 | GU347728 | GU348072 |
|  | *Amara anthobia* A. & G.B. Villa, 1833 | ZFMK_COL_2008_86 | Waltrop, NRW, GER | GU347041 | GU347385 | GU347729 | GU348073 |
|  | *Amara aulica* (Panzer, 1797) | ZFMK_COL_2008_151 | Waltrop, NRW, GER | GU347042 | GU347386 | GU347730 | GU348074 |
|  | *Amara aulica* (Panzer, 1797) | ZFMK_COL_2008_205 | Waltrop, NRW, GER | GU347043 | GU347387 | GU347731 | GU348075 |
|  | *Amara erratica* (Duftschmid, 1812) | ZFMK_COL_2009_553 | Zillertaler Alpen, TI, AU | GU347044 | GU347388 | GU347732 | GU348076 |
|  | *Amara erratica* (Duftschmid, 1812) | ZFMK_COL_2009_554 | Zillertaler Alpen, TI, AU | GU347046 | GU347390 | GU347734 | GU348078 |
|  | *Amara erratica* (Duftschmid, 1812) | ZFMK_COL_2009_555 | Zillertaler Alpen, TI, AU | GU347047 | GU347391 | GU347735 | GU348079 |
|  | *Amara erratica* (Duftschmid, 1812) | ZFMK_COL_2009_556 | Zillertaler Alpen, TI, AU | GU347048 | GU347392 | GU347736 | GU348080 |
|  | *Amara erratica* (Duftschmid, 1812) | ZFMK_COL_2009_557 | Zillertaler Alpen, TI, AU | GU347045 | GU347389 | GU347733 | GU348077 |
|  | *Amara quenseli* (Schönherr, 1806) | ZFMK_COL_2009_531 | Zillertaler Alpen, TI, AU | GU347049 | GU347393 | GU347737 | GU348081 |
|  | *Amara quenseli* (Schönherr, 1806) | ZFMK_COL_2009_532 | Zillertaler Alpen, TI, AU | GU347051 | GU347395 | GU347739 | GU348083 |
|  | *Amara quenseli* (Schönherr, 1806) | ZFMK_COL_2009_533 | Zillertaler Alpen, TI, AU | GU347052 | GU347396 | GU347740 | GU348084 |
|  | *Amara quenseli* (Schönherr, 1806) | ZFMK_COL_2009_534 | Zillertaler Alpen, TI, AU | GU347053 | GU347397 | GU347741 | GU348085 |
|  | *Amara quenseli* (Schönherr, 1806) | ZFMK_COL_2009_535 | Zillertaler Alpen, TI, AU | GU347050 | GU347394 | GU347738 | GU348082 |
|  | *Amara similata* (Gyllenhal, 1810) | ZFMK_COL_2008_163 | Waltrop, NRW, GER | GU347054 | GU347398 | GU347742 | GU348086 |
|  | *Amara similata* (Gyllenhal, 1810) | ZFMK_COL_2008_175 | Waltrop, NRW, GER | GU347056 | GU347400 | GU347744 | GU348088 |
|  | *Amara similata* (Gyllenhal, 1810) | ZFMK_COL_2008_459 | Fehmarn, SH, GER | GU347055 | GU347399 | GU347743 | GU348087 |
| Harpalini | *Anisodactylus binotatus* (Fabricius, 1787) | ZFMK_COL_2008_190 | Waltrop, NRW, GER | GU347063 | GU347407 | GU347751 | GU348095 |
|  | *Anisodactylus binotatus* (Fabricius, 1787) | ZFMK_COL_2008_471 | Waltrop, NRW, GER | GU347064 | GU347408 | GU347752 | GU348096 |
|  | *Harpalus affinis* (Schrank, 1781) | ZFMK_COL_2008_425 | Fehmarn, SH, GER | GU347214 | GU347558 | GU347902 | GU348246 |
|  | *Harpalus affinis* (Schrank, 1781) | ZFMK_COL_2008_426 | Fehmarn, SH, GER | GU347216 | GU347560 | GU347904 | GU348248 |
|  | *Harpalus affinis* (Schrank, 1781) | ZFMK_COL_2008_435 | Dresden, S, GER | GU347217 | GU347561 | GU347905 | GU348249 |
|  | *Harpalus affinis* (Schrank, 1781) | ZFMK_COL_2008_436 | Dresden, S, GER | GU347218 | GU347562 | GU347906 | GU348250 |
|  | *Harpalus affinis* (Schrank, 1781) | ZFMK_COL_2008_437 | Dresden, S, GER | GU347219 | GU347563 | GU347907 | GU348251 |
|  | *Harpalus affinis* (Schrank, 1781) | ZFMK_COL_2009_619 | Teutschenthal, SA, GER | GU347220 | GU347564 | GU347908 | GU348252 |
|  | *Harpalus affinis* (Schrank, 1781) | ZFMK_COL_2009_620 | Teutschenthal, SA, GER | GU347221 | GU347565 | GU347909 | GU348253 |
|  | *Harpalus affinis* (Schrank, 1781) | ZFMK_COL_2009_621 | Teutschenthal, SA, GER | GU347222 | GU347566 | GU347910 | GU348254 |
|  | *Harpalus affinis* (Schrank, 1781) | ZFMK_COL_2009_622 | Teutschenthal, SA, GER | GU347215 | GU347559 | GU347903 | GU348247 |
|  | *Harpalus rubripes* (de Geer, 1774) | ZFMK_COL_2008_428 | Fehmarn, SH, GER | GU347223 | GU347567 | GU347911 | GU348255 |
|  | *Harpalus rubripes* (de Geer, 1774) | ZFMK_COL_2009_218 | Castrop-Rauxel, NRW, GER | GU347224 | GU347568 | GU347912 | GU348256 |
|  | *Harpalus rubripes* (de Geer, 1774) | ZFMK_COL_2009_219 | Castrop-Rauxel, NRW, GER | GU347225 | GU347569 | GU347913 | GU348257 |
|  | *Harpalus rufipes* (de Geer, 1774) | ZFMK_COL_2008_273 | Waltrop, NRW, GER | GU347226 | GU347570 | GU347914 | GU348258 |
|  | *Harpalus rufipes* (de Geer, 1774) | ZFMK_COL_2009_222 | Schmedehausen, NRW, GER | GU347228 | GU347572 | GU347916 | GU348260 |
|  | *Harpalus rufipes* (de Geer, 1774) | ZFMK_COL_2009_602 | Remagen, RP, GER | GU347227 | GU347571 | GU347915 | GU348259 |
|  | *Dicheirotrichus gustavii* Crotch, 1871 | ZFMK_COL_2008_28 | Dangast, NS, GER | GU347172 | GU347516 | GU347860 | GU348204 |
|  | *Dicheirotrichus gustavii* Crotch, 1871 | ZFMK_COL_2008_29 | Dangast, NS, GER | GU347173 | GU347517 | GU347861 | GU348205 |
|  | *Dicheirotrichus gustavii* Crotch, 1871 | ZFMK_COL_2008_30 | Dangast, NS, GER | GU347174 | GU347518 | GU347862 | GU348206 |
|  | *Dicheirotrichus gustavii* Crotch, 1871 | ZFMK_COL_2008_31 | Dangast, NS, GER | GU347175 | GU347519 | GU347863 | GU348207 |
|  | *Dicheirotrichus gustavii* Crotch, 1871 | ZFMK_COL_2008_32 | Dangast, NS, GER | GU347176 | GU347520 | GU347864 | GU348208 |
|  | *Dicheirotrichus obsoletus* (Dejean, 1829) | ZFMK_COL_2009_425 | Halle an der Saale, SA, GER | GU347177 | GU347521 | GU347865 | GU348209 |
|  | *Dicheirotrichus obsoletus* (Dejean, 1829) | ZFMK_COL_2009_426 | Halle an der Saale, SA, GER | GU347178 | GU347522 | GU347866 | GU348210 |
|  | *Dicheirotrichus obsoletus* (Dejean, 1829) | ZFMK_COL_2009_427 | Halle an der Saale, SA, GER | GU347179 | GU347523 | GU347867 | GU348211 |
|  | *Dicheirotrichus obsoletus* (Dejean, 1829) | ZFMK_COL_2009_428 | Halle an der Saale, SA, GER | GU347180 | GU347524 | GU347868 | GU348212 |
|  | *Stenolophus mixtus* (Herbst, 1784) | ZFMK_COL_2008_78 | Waltrop, NRW, GER | GU347346 | GU347690 | GU348034 | GU348378 |
|  | *Stenolophus mixtus* (Herbst, 1784) | ZFMK_COL_2008_81 | Waltrop, NRW, GER | GU347347 | GU347691 | GU348035 | GU348379 |
|  | *Stenolophus teutonus* (Schrank, 1781) | ZFMK_COL_2008_87 | Waltrop, NRW, GER | GU347352 | GU347696 | GU348040 | GU348384 |
|  | *Stenolophus teutonus* (Schrank, 1781) | ZFMK_COL_2009_487 | Bienen, NRW, GER | GU347348 | GU347692 | GU348036 | GU348380 |
|  | *Stenolophus teutonus* (Schrank, 1781) | ZFMK_COL_2009_488 | Bitterfeld, SA, GER | GU347349 | GU347693 | GU348037 | GU348381 |
|  | *Stenolophus teutonus* (Schrank, 1781) | ZFMK_COL_2009_627 | Bienen, NRW, GER | GU347350 | GU347694 | GU348038 | GU348382 |
|  | *Stenolophus teutonus* (Schrank, 1781) | ZFMK_COL_2009_628 | Bienen, NRW, GER | GU347351 | GU347695 | GU348039 | GU348383 |
| Licinini | *Licinus hoffmannseggii* (Panzer, 1803) | ZFMK_COL_2009_579 | Zillertaler Alpen, TI, AU | GU347229 | GU347573 | GU347917 | GU348261 |
|  | *Licinus hoffmannseggii* (Panzer, 1803) | ZFMK_COL_2009_580 | Zillertaler Alpen, TI, AU | GU347231 | GU347575 | GU347919 | GU348263 |
|  | *Licinus hoffmannseggii* (Panzer, 1803) | ZFMK_COL_2009_581 | Zillertaler Alpen, TI, AU | GU347232 | GU347576 | GU347920 | GU348264 |
|  | *Licinus hoffmannseggii* (Panzer, 1803) | ZFMK_COL_2009_586 | Totes Gebirge, SM, AU | GU347230 | GU347574 | GU347918 | GU348262 |
| Lebiini | *Dromius quadrimaculatus* (Linne, 1758) | ZFMK_COL_2008_134 | Waltrop, NRW, GER | GU347181 | GU347525 | GU347869 | GU348213 |
|  | *Dromius quadrimaculatus* (Linne, 1758) | ZFMK_COL_2009_178 | Fehmarn, SH, GER | GU347182 | GU347526 | GU347870 | GU348214 |
|  | *Dromius quadrimaculatus* (Linne, 1758) | ZFMK_COL_2009_179 | Fehmarn, SH, GER | GU347183 | GU347527 | GU347871 | GU348215 |
|  | *Philorhizus melanocephalus* (Linne, 1758) | ZFMK_COL_2009_269 | Schmedehausen, NRW, GER | GU347269 | GU347613 | GU347957 | GU348301 |
|  | *Philorhizus melanocephalus* (Linne, 1758) | ZFMK_COL_2009_270 | Fehmarn, SH, GER | GU347270 | GU347614 | GU347958 | GU348302 |
|  | *Philorhizus melanocephalus* (Linne, 1758) | ZFMK_COL_2009_271 | Fehmarn, SH, GER | GU347271 | GU347615 | GU347959 | GU348303 |
|  | *Philorhizus sigma* (Rossi, 1790) | ZFMK_COL_2009_272 | Fehmarn, SH, GER | GU347272 | GU347616 | GU347960 | GU348304 |
|  | *Philorhizus sigma* (Rossi, 1790) | ZFMK_COL_2009_273 | Fehmarn, SH, GER | GU347273 | GU347617 | GU347961 | GU348305 |
