# Supplementary material for: Molecular species identification of Central European ground beetles (Coleoptera: Carabidae) using nuclear rDNA expansion segments and DNA barcodes
Source: Front Zool. 2010 Sep 13;7:26. doi: 10.1186/1742-9994-7-26 (PMC2945340; doi:10.1186/1742-9994-7-26)
Supplement: Additional file 6 — Primers and PCR protocols used in this study. [file 1742-9994-7-26-S6.DOC]

**Used PCR and sequencing primers:**

| **Name of DNA marker and primer** | **Primer sequence (5’ to 3’)** | **Reference** |
| --- | --- | --- |
|  |  |  |
| **Cytochrome *c* oxidase subunit I** |  |  |
| LCO-1490 | GGTCAACAAATCATAAAGATATTGG | Folmer et al. 1994 |
| HCO-2198 | TAAACTTCAGGGTGACCAAAAAATCA | Folmer et al. 1994 |
|  |  |  |
| **18S rDNA: V4** |  |  |
| CV4F | TGGTGCCAGCAGCCGCGGTAA | This study |
| CV4R | CCTCTAACGTCGCAATACGAATGCCC | This study |
|  |  |  |
| **18S rDNA: V7** |  |  |
| CV7F | CTTAAAGGAATTGACGGAGGGCACCACC | This study |
| CV7R | GATTCCTTCAGTGTAGCGCGCGTG | This study |
|  |  |  |
| **28S rDNA: D3** |  |  |
| CD3F | GGACCCGTCTTGAAACAC | This study |
| CD3R | GCATAGTTCACCATCTTTC | This study |

**Used PCR profiles:**

**Cytochrome *c* oxidase subunit I**

Initial denaturation: 94°C (5 min)

Amplification (38x)

Denaturation: 94°C (45 s)

Annealing: 48°C (45 s)

Extension: 72°C (80 s)

Final extension: 72°C (7 min)

**18S rDNA: V4**

Initial denaturation: 94°C (5 min)

Amplification (32x)

Denaturation: 94°C (45 s)

Annealing: 66°C (45 s)

Extension: 72°C (2 min)

Final extension: 72°C (8 min)

**18S rDNA: V7**

Initial denaturation: 94°C (5 min)

Amplification (32x)

Denaturation: 94°C (45 s)

Annealing: 68°C (45 s)

Extension: 72°C (2 min)

Final extension: 72°C (8 min)

**28S rDNA: D3**

Initial denaturation: 94°C (5 min)

Amplification (32x)

Denaturation: 94°C (45 s)

Annealing: 52°C (45 s)

Extension: 72°C (80 s)

Final extension: 72°C (7 min)
