# Supplementary material for: Molecular species identification of Central European ground beetles (Coleoptera: Carabidae) using nuclear rDNA expansion segments and DNA barcodes
Source: Front Zool. 2010 Sep 13;7:26. doi: 10.1186/1742-9994-7-26 (PMC2945340; doi:10.1186/1742-9994-7-26)
Supplement: Additional file 7 — Order of species used for the Klee diagram of indicator vector correlations of COI sequences. The analysis is based on a Neighbor-joining (NJ) analysis using p-distances. [file 1742-9994-7-26-S7.DOC]

| **Number** | **Species** |
| --- | --- |
| 1 | *Pterostichus rhaeticus* |
| 2 | *Pterostichus nigrita* |
| 3 | *Pterostichus anthracinus* |
| 4 | *Philorhizus sigma* |
| 5 | *Philorhizus melanocephalus* |
| 6 | *Pterostichus unctulatus* |
| 7 | *Pterostichus melanarius* |
| 8 | *Poecilus cupreus* |
| 9 | *Amara aulica* |
| 10 | *Pterostichus aterrimus* |
| 11 | *Pterostichus oblongopunctatus* |
| 12 | *Pterostichus illigeri* |
| 13 | *Pterostichus niger* |
| 14 | *Pterostichus jurinei* |
| 15 | *Pterostichus ziegleri* |
| 16 | *Pterostichus panzeri* |
| 17 | *Agonum marginatum* |
| 18 | *Agonum muelleri* |
| 19 | *Anchomenus dorsalis* |
| 20 | *Agonum micans* |
| 21 | *Agonum emarginatum* |
| 22 | *Agonum viduum* |
| 23 | *Amara similata* |
| 24 | *Amara anthobia* |
| 25 | *Amara erratica* |
| 26 | *Anisodactlyus binotatus* |
| 27 | *Harpalus rufipes* |
| 28 | *Harpalus rubripes* |
| 29 | *Harpalus affinis* |
| 30 | *Dromius quadrimaculatus* |
| 31 | *Elaphrus cupreus* |
| 32 | *Elaphrus aureus* |
| 33 | *Elaphrus riparius* |
| 34 | *Limodromus assimilis* |
| 35 | *Amara quenseli* |
| 36 | *Cychrus caraboides* |
| 37 | *Carabus nitens* |
| 38 | *Carabus monilis* |
| 39 | *Carabus nemoralis* |
| 40 | *Carabus auronitens* |
| 41 | *Elaphropus quadrisignatus* |
| 42 | *Elaphropus pavulus* |
| 43 | *Dicheirotrichus obsoletus* |
| 44 | *Dicheirotrichus gustavii* |
| 45 | *Stenolophus mixtus* |
| 46 | *Stenolophus teutonus* |
| 47 | *Dyschirius aeneus* |
| 48 | *Dyschirius chalceus* |
| 49 | *Dyschirius thoracicus* |
| 50 | *Omophron limbatum* |
| 51 | *Molops piceus* |
| 52 | *Licinus hoffmannseggii* |
| 53 | *Abax beckenhauptii* |
| 54 | *Abax parallelepipedus* |
| 55 | *Abax ovalis* |
| 56 | *Clivina collaris* |
| 57 | *Clivina fossor* |
| 58 | *Loricera pilicornis* |
| 59 | *Bembidion ruficorne* |
| 60 | *Bembidion decoratum* |
| 61 | *Bembidion elongatum* |
| 62 | *Nebria jockischii* |
| 63 | *Nebria hellwigii* |
| 64 | *Oreonebria castanea* |
| 65 | *Bembidion punctulatum* |
| 66 | *Bembidion pallidipenne* |
| 67 | *Bembidion tibiale* |
| 68 | *Bembidion tetracolum* |
| 69 | *Bembidion decorum* |
| 70 | *Bembidion articulatum* |
| 71 | *Bembidion aspericolle* |
| 72 | *Bembidion semipunctatum* |
| 73 | *Bembidion litorale* |
| 74 | *Bembidion properans* |
| 75 | *Bembidion lampros* |
